# A new low-turbulence wind tunnel for animal and small vehicle flight experiments

**DOI:** 10.1098/rsos.160960

**Published:** 2017-03-29

**Authors:** Daniel B. Quinn, Anthony Watts, Tony Nagle, David Lentink

**Affiliations:** 1 Department of Mechanical Engineering, Stanford University, Stanford, CA, USA; 2 Jacobs, Tullahoma, TN, USA

**Keywords:** biomechanics, wind tunnel, turbulence, unmanned aerial vehicles, flight stability, gust mitigation

## Abstract

Our understanding of animal flight benefits greatly from specialized wind tunnels designed for flying animals. Existing facilities can simulate laminar flow during straight, ascending and descending flight, as well as at different altitudes. However, the atmosphere in which animals fly is even more complex. Flow can be laminar and quiet at high altitudes but highly turbulent near the ground, and gusts can rapidly change wind speed. To study flight in both laminar and turbulent environments, a multi-purpose wind tunnel for studying animal and small vehicle flight was built at Stanford University. The tunnel is closed-circuit and can produce airspeeds up to 50 m s^−1^ in a rectangular test section that is 1.0 m wide, 0.82 m tall and 1.73 m long. Seamless honeycomb and screens in the airline together with a carefully designed contraction reduce centreline turbulence intensities to less than or equal to 0.030% at all operating speeds. A large diameter fan and specialized acoustic treatment allow the tunnel to operate at low noise levels of 76.4 dB at 20 m s^−1^. To simulate high turbulence, an active turbulence grid can increase turbulence intensities up to 45%. Finally, an open jet configuration enables stereo high-speed fluoroscopy for studying musculoskeletal control in turbulent flow.

## 1. Introduction

Wind tunnels are essential tools for studying both vehicle and animal flight in laminar and turbulent flows. The key advantage of wind tunnels for biological research is that airspeed can be finely controlled over an animal that remains stationary in the laboratory frame. This fixed frame allows direct measurements of forces, kinematics and flow fields that would otherwise be unattainable. Inspired by these advantages, several laboratories have built open-circuit wind tunnels designed for studying bird flight in the past 50 years [1–9]. Open-circuit tunnels typically produce moderate turbulent intensities (1–3%) and therefore provide valuable information about animal flight in moderate turbulence. Flight environments, however, span a wide range of turbulence, from the extremely laminar flows found in the upper atmosphere (less than 0.1% [10,11]) to the extremely turbulent flows found in forests and street canyons (10–40% [12–14]). Turbulence significantly affects flight performance, because it promotes boundary layer transition and affects lift/drag measurements, especially if lift-generating surfaces are operating near stall [15]. As a result, turbulence has been shown to decrease the flight speed of bees [16,17] and moths [18] and increase flight costs in hummingbirds [19].

To study flight in extremely laminar environments, some laboratories have built specialized closed-circuit wind tunnels with very low turbulence levels. While modern open-circuit tunnels can produce turbulence intensities as low as 0.1% [20], they cannot achieve the extremely low turbulence and temperature control possible in closed-circuit tunnels. The Royal Institute of Technology (KTH) [21,22] and Texas A&M [23,24], for example, have closed-circuit tunnels with axial turbulence intensities of less than 0.040% and less than 0.047%, respectively (measured at 10 m s^−1^). A look at the properties of current state-of-the-art animal flight tunnels shows the trade-offs associated with low-turbulence tunnel design (table 1). The tunnel at the University of Western Ontario produces higher turbulence intensities (less than 0.3%) but is able to simulate flight at altitudes up to 7 km [25,26]. The tunnel at Lund University produces low turbulence (less than 0.06%) and tilts to simulate ascending (up to 8°) and descending (up to 6°) flight [27–29]. None of these tunnels, however, can produce both highly laminar and highly turbulent flows.

**Table 1.** Recent closed-circuit wind tunnels produce low turbulence intensities. Modern closed-circuit tunnels use a combination of honeycombs, screens and contractions to create extremely low turbulence intensities. Axial turbulence intensity is the root mean square fluctuation in streamwise velocity compared to the bulk airspeed. Measurements have been reported with different methods and positions within wind tunnels, so identical conditions for comparison are not available in every case. The grid over which measurements were made also varies; the grid at KTH [22], for example, includes points closer to the wall than those reported here. Where possible, tunnels were compared at airspeeds relevant for animal flight (approx. 10 m s^−1^). n.a., no data available.

	location	test section w × h × l (m)	contraction ratio	max speed (m s^–1^)	axial turbulence intensity (%)	additional features
open circuit	Harvard U. [4]	1.2 × 1.2 × 1.4	6	28.5	<1.28^a^	animal flight
	U. Montana [6]	0.6 × 0.6 × 0.85	6	12	1.2^b^	animal flight
	U. Birmingham [5]	2.5 × 2.1 × 3.1	n.a.	n.a.	∼0.8^c^	animal flight
	U. Illinois [20]	0.9 × 1.2 × 2.4	7.5	71.5	∼0.08^d^	
closed circuit	Saarland U. [7,8]	1 × 1 × 1	n.a.	14	∼2^e^	animal flight
	U. Western Ontario [25,26]	1.5 × 1 × 2	2.5	21	<0.3^f^	animal flight
						hypobaric
						temp. control
						hum. control
	Lund U. [27–29]	1.2 × 1.1 × 1.7	12.25	38	<0.06^g^	animal flight
						tilting section
						temp. control
	U. Southern California [30]	1.4 × 1.4 × 2.1	n.a.	n.a.	∼0.025^h^	
	Texas A&M U. [23,24]	1.4 × 1.4 × 4.9	5.33	31	0.047^i^	
	KTH [21,22]	1.2 × 0.8 × 7	9	69	<0.040^j^	temp. control
	Stanford	1 × 0.8 × 1.7	7	50	<0.030^j^	animal flight
						open/closed-jet
						turb. control
						temp. control

^a^Maximum value across test section at 10 m s^−1^ using the turbulence sphere method [31].

^b^Centreline value between 6 and 18 m s^−1^ using the turbulence sphere method [31].

^c^Centreline value at 17.9 m s^−1^, filter type not published.

^d^Centreline value at 9.8 m s^−1^ with a 0.1 Hz to 5 kHz bandpass filter.

^e^Value 25 cm from one corner between 0 and 14 m s^−1^ with a 0.5 Hz to 1 kHz bandpass filter.

^f^Conditions not published.

^g^Maximum value across test section at 10 m s^−1^ using 1.024 s samples collected at 1 kHz.

^h^Maximum value across test section at 7 m s^−1^ with a 2–200 Hz bandpass filter.

^i^Centreline value at 10 m s^−1^ with a 1 Hz to 10 kHz bandpass filter.

^j^Maximum value across test section at 10 m s^−1^ with a 20 Hz highpass filter.

To advance the capabilities of animal flight wind tunnels for biomechanics and aerodynamics studies, we built a multi-purpose tunnel that produces low streamwise turbulence (less than or equal to 0.028% at the centreline over the full operating range), but also high turbulence using an active grid of spinning vanes (up to approx. 45% at the centreline). The low turbulence levels are made possible by six seamless screens, a seamless honeycomb and a 7 : 1 contraction ratio. The tunnel is also barely audible at low airspeeds. Measured noise levels in the test section reach 76.4 dB at 20 m s^−1^, allowing quiet natural flight conditions for animals and minimal acoustic disturbances to the flow. The low noise is a result of acoustic treatment throughout the tunnel and an acoustic wall that separates the large diameter fan from the testing area. The tunnel test section (1.0 m wide, 0.82 m tall and 1.73 m long) can be removed to operate the tunnel with an open jet of air in the testing area, allowing the placement of large diagnostic equipment, such as biplanar fluoroscopes for high-speed musculoskeletal recordings. A water-chilled heat exchanger keeps the test section temperature steady (1*σ* = 0.007°C at 10 m s^−1^) over a range of 10–30°C. The tunnel is washable, and all sections are accessible via hatches, making the tunnel easy to keep sanitary for animals. A system of redundant screens and netting keeps animals safely within the testing area. The high-quality flow and the special features of the multi-purpose tunnel make it the first of its kind. Here, we give a complete description of its layout and its aeroacoustic performance. To define the performance metrics for the wind tunnel, we used the KTH MTL wind tunnel as our primary reference, because it performs well and is relatively well documented [21,22].

## 2. Tunnel layout

**Figure 1.**
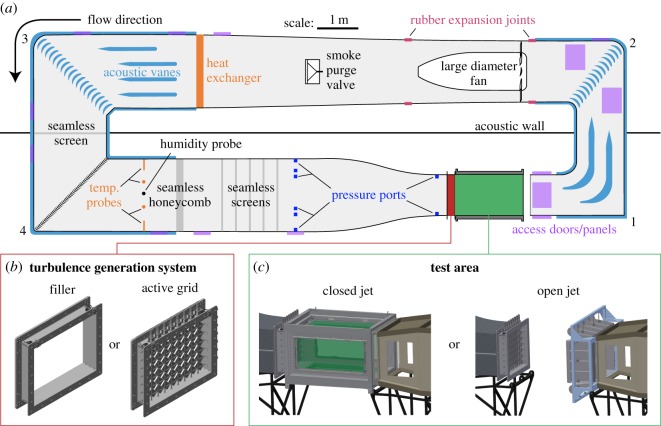
The closed-circuit wind tunnel uses flow conditioning to decrease turbulence and can operate in both open- and closed-jet configurations. (*a*) The components of the wind tunnel are shown by colour. A large diameter fan drives air through a series of flow conditioning elements, then through a contraction into the test section (green). A heat exchanger and temperature probes (orange) are used to regulate temperature in the tunnel. Airspeed is measured using static pressure ports in the stilling chamber and contraction (dark blue). The turning vanes and sidewalls shown in light blue are filled with mineral wool to attenuate fan noise. To facilitate discussion in the text, the corners are labelled 1–4 as shown. Electronic supplementary material, figure SF.1 shows a photograph of the tunnel taken from within the laboratory. (*b*) Just upstream of the test section, an active turbulence grid can be positioned to inject turbulence into the flow. For low-turbulence experiments, a filler with identical dimensions is used instead. (*c*) The test section can be removed, and collector flaps can be installed on the inflow of corner 1. The resulting ‘open-jet’ configuration allows easy access to an exposed jet of air in the testing area.

The new wind tunnel is a closed-circuit tunnel with a footprint of 15.1 × 5.2 m and a maximum height of 4 m. The components of the tunnel are designed to work together to create flow with uniform speed, uniform temperature, low turbulence and a low noise profile (figure 1).

### 2.1. Fan

The airflow is driven by a single stage, 18 blade, axial fan. The fan was selected based on its low acoustic signature, which helped to minimize acoustic levels in the test section. A 1.5 m diameter fan was chosen to maintain the compact size of the wind tunnel while meeting the wind tunnel's low noise requirements. To reduce vibration transmission, the fan's steel housing was mounted on its own massive concrete foundation, separate from the building foundation and connected to the tunnel through expansion joints. The fan is powered by an 80 kW induction motor (891 r.p.m. max speed; ±0.1 r.p.m. resolution), which is housed in an enclosed nacelle. A tail-cone is provided to further improve fan efficiency as well as to provide good airflow performance downstream. The motor is water-cooled and controlled by a variable frequency drive which uses a 100 : 1 turndown ratio to regulate fan speed based on user input. The drive-motor assembly is equipped with braking in the event of an emergency stop.

### 2.2. Flow conditioning

A series of flow conditioning elements are used to straighten and laminarize the flow before it enters the test section. Turning vanes were designed to straighten the flow while minimizing induced turbulence and circuit pressure loss. A honeycomb in the stilling chamber (cell length/diameter = 16) straightens the flow before it enters the flow conditioning screens. To maximize flow alignment, the honeycomb was made seamless and uniform by welding together precisely crimped stainless steel strips. Downstream of the honeycomb, five seamless, fine mesh, stainless steel screens are used to reduce turbulence in the stilling chamber. The mesh screens are spaced 350 mm apart have wire diameters of 0.16 mm, nominal apertures of 0.5 × 0.5 mm and open areas of 57.4%. A sixth identical screen is located in the crossleg between corners 3 and 4 for additional turbulence reduction. Each of the screens is pre-tensioned to reduce sag and lateral displacement. To prevent flow perturbations in the stilling chamber, fairing plates were used to conceal the screen and honeycomb attachment points. Following the honeycomb and flow conditioning screens in the stilling chamber, the flow passes through a 7 : 1 fibreglass contraction, which reduces turbulence intensity by accelerating the flow. The contraction length and curvature were designed with computational fluid dynamics (CFD) simulations to reduce velocity gradients at the contraction exit, thereby minimizing the anisotropy of turbulence in the test section.

### 2.3. Testing area

The testing area can be operated in closed jet, with the test section installed, or open jet, with the test section removed and collector flaps installed (figure 1*c*).

The closed-jet configuration allows maximum airspeeds with minimal turbulence, flow angularity and pressure variation. When operating in closed-jet configuration, a black anodized aluminium test section (airline dimensions 1.0 m wide, 0.82 m tall and 1.73 m long) connects the contraction to corner 1. A breather gap downstream of the test section assures that testing pressures are near atmospheric, allowing access for probes and supports without the need for airtight access ports. Streamwise gradients in pressure are minimized by diverging test section walls that account for boundary layer growth. The floor and ceiling divergence angle is fixed (0.5°) while the sidewall divergence angle is variable to adjust for different blockage levels (see the electronic supplementary material, SM.1).

The test section is designed for efficient high-fidelity diagnostics on animal and small vehicle flight. All components in and around the test section are matt black to facilitate high-speed videography and particle tracking. In addition, each wall of the test section contains a panel that can be removed for repair, replacement, modification or for quick access to the test section interior. Two sets of side panels were built: translucent acrylic, designed for durability during training flights; and thin optical glass, designed for the high transparency required for laser-based flow measurements. The area downstream of the test section is equipped with side and floor hatches (with windows) for easy access while working with animals (electronic supplementary material, figure SF.10).

The open-jet configuration allows even easier access to live animals during training sessions in the airflow. It also allows large diagnostic machinery, such as biplanar fluoroscopy emitters and receivers, to be positioned around the flow. To switch into open-jet mode, the test section (approx. 270 kg) is removed with a hydraulic cart, and matt black aluminium collector flaps are attached to corner 1. The angle of the flaps can be adjusted to maximize pressure recovery and minimize flow divergence.

### 2.4. Airspeed control

Airspeeds up to 50 m s^−1^ in the test section are determined by measuring static pressure readings in the stilling chamber and the contraction. For both readings, pressure is mechanically averaged over two sets of four permanently installed static pressure ports. One set of ports from each section connects to a differential pressure sensor (Rosemount 3051S) tuned to measure airspeeds from 0 to 15 m s^−1^. The other set connects to a differential pressure sensor tuned to measure airspeeds from 15–50 m s^−1^. This multi-sensor approach was chosen to ensure high-precision airspeed control at both low and high speeds. A third pressure sensor calculates gauge pressure in the contraction by comparing the static pressure to atmospheric pressure. During commissioning, a Pitot-static probe (Kanomax 1 m) connected to a high-accuracy pressure transducer (MKS 690A/698A) was placed at the centreline in the test section. A second-order polynomial curve fit was generated between the facility pressure port readings and the readings from the Pitot-static probe. This curve fit is used to correct the facility pressure port readings to the true dynamic and static pressures in the test section. Airspeed is calculated from these corrected pressures by using the isentropic flow equations for an ideal gas, including correction factors for temperature and humidity (electronic supplementary material, SM.2).

The airspeed is controlled in open loop by setting the fan speed (r.p.m.), which is determined from a user-defined airspeed via a lookup table. The default fan speed lookup table for an empty test section was calibrated with the Pitot-static probe during commissioning. This lookup table can be modified when blockage levels change—such as when operating in open jet—where the mapping from fan speed to airspeed is different. The lookup tables are updated through a custom Human Machine Interface written in LabVIEW that sends commands via Ethernet to a Programmable Logic Controller. Once the table has been uploaded, a user can enter an airspeed set point into the Human Machine Interface for automated airspeed control.

### 2.5. Temperature control

The temperature in the tunnel is controlled by a Proportional-Integral controller that regulates input to a water-cooled heat exchanger. The controller compares a user-defined set point temperature to the average reading of four resistance thermometers in corner 4. Based on this comparison, the controller operates two control valves that regulate the exchange of chilled water (approx. 7°C) with water pumped at constant rate through the heat exchanger. This use of a small chilled water loop to regulate the primary coolant loop was chosen to simplify the controller and thus reduce time to reach thermal equilibrium. The controller gains are automatically adjusted based on airspeed to ensure thermal equilibrium at all speeds. Because the tunnel is in a climate-controlled laboratory, a separate heater was not necessary; ambient heat and friction losses in the fan are sufficient to warm the tunnel to the airspeed-dependent temperature range (electronic supplementary material, figure SF.11).

The heat exchanger was designed to maximize water–air heat transfer, maximize temperature uniformity and minimize turbulence generation. The exchanger consists of two sets of vertical copper tubes connected to thin continuous horizontal aluminium fins, all of which are housed in an insulated galvanized steel frame to eliminate oxidation at the edges. To increase temperature uniformity, water is pumped at a constant flow rate and the flow direction reverses between the two sets of tubes. The exchanger is positioned just downstream of the diffuser following the fan. This position was chosen for three reasons: (i) the resulting back pressure helps to keep the flow attached in the diffuser, (ii) any resulting turbulence has time to decay before reaching the test section and (iii) the resulting noise and pressure drop are minimized by placing the exchanger in a section with wide area and thus low airspeed (predicted maximum of 7.1 m s^−1^).

### 2.6. Active turbulence grid

While the tunnel is designed to produce extremely laminar flow, it is also capable of simulating the highly turbulent conditions that animals and vehicles encounter in the atmospheric boundary layer. High turbulence is introduced with the use of an active grid of spinning diamond-shaped vanes, a concept introduced by Makita & Sassa [32]. Our grid in particular was modelled after a similar system developed by Cekli *et al*. [33,34]. The grid is made up of 7 rows and 8 columns of vanes, which are actuated independently by 150 W DC motors (Maxon RE40). For low turbulence conditions, the grid of vanes can be replaced by an empty filler section (figure 1*b*).

The active grid of vanes can also be used to simulate non-uniform flows in the testing area. For example, by adjusting the fixed position of the vanes, a jet or wake profile can be created downstream of the grid. This technique could be used to investigate flight in turbulent wakes or jets and compare the results with low turbulence conditions. The positions of the vanes can be controlled automatically based on high-speed marker tracking. This enables the grid to react in real time to the kinematics of animals or vehicles flying in the test area.

### 2.7. Additional features

*Expanding/contracting corners*. To limit the overall circuit length while maintaining flow quality, corners 1 and 2 expand with expansion ratios of 1.17 and 1.28, respectively. The corresponding diffusion half-angles are 1.5° and 2.6°. The heat exchanger pressure drop immediately downstream of the backleg diffuser was included in the design to prevent flow separation at the expansion points. The values for the expansion ratio were chosen to minimize the tunnel footprint while maintaining flow uniformity and minimizing fan power consumption. Similar expansion ratios have been used in previous successful designs by Jacobs Engineering and were verified via CFD for the present wind tunnel.

*Acoustic treatment*. Acoustic material is used throughout the tunnel to create a low-noise environment in the test section. The internal side walls of the corners and crosslegs are lined with mineral wool (100 mm thickness) encased in perforated sheet metal, designed to attenuate sound in the mid-to-high frequency range. The turning vanes in corners 1–3 are filled with mineral wool as well. The vanes in corner 1 are also covered with acoustically transparent carbon fibre cloth that redirects the flow smoothly while maintaining the acoustic absorption properties of the vane. For maximum noise reduction, synthetic washable fur covers can be placed over the leading edges of the corner 1 turning vanes.

*Cleaning and human safety*. To maintain sanitary conditions for animals, the tunnel was designed such that it can be washed efficiently. All surfaces—both external and internal—can be cleaned with a low-pressure wash of diluted bleach, which drains through ports in the tunnel floor. The purpose of the wash is to remove flow seeding residue, animal faecal matter and/or feathers. A pulley system slides out the turbulence screens for easy cleaning access. Ceiling panels are designed to support approximately 100 kg for easy access to upper exterior sections and installation of equipment, and all internal sections are accessible through hatches on the sides or bottom of the tunnel. An interlock safety system prevents the tunnel from running when hatches near the fan are open. Finally, the entire wind tunnel laboratory is lead-shielded to meet X-ray health and safety standards for high-speed biplanar fluoroscopy.

*Animal safety*. A series of protective screens, laser-curtains and nets keeps animals confined to the testing area. First, an array of vertical steel strings (0.5 mm diameter, 10 mm spacing) prevents animals from flying upstream of the test section. Tensioned music wire (high-carbon steel) was chosen because of its high tensile strength and stiffness. These properties (i) minimize flow disturbance by ensuring that the strings are as straight and thin as possible and (ii) minimize wire damage due to beaks and/or claws. Vertical wires were chosen to prevent animals from perching on the strings. The strings are matt black to facilitate high-speed particle tracking and avoid unwanted visual cues for animals flying in the test section. After birds are trained to fly reliably in the test section without entering the pitch-black settling chamber, the array of wires can be removed for experiments where minimal turbulence is required. Likewise, engineering aerodynamic research will be performed without the array of wires. Therefore, the turbulence intensities reported here were measured with the array removed. Flexible netting was installed downstream of the test section and around the breather gap. Together, the wire array and the netting keep animals safely confined to the testing area. In addition, a curtained antechamber around the tunnel and a permanent metal grid between corners 1 and 2 provide failsafe layers of secondary protection for animals. Finally, the laboratory can be hosed down, the air volume is refreshed 15 times per hour, and all lights are flicker-free to meet all modern animal research standards.

*Humidity measurement*. While relative humidity is not actively controlled, it is measured by a humidity sensor downstream of corner 4 and was found to stay relatively constant (±0.08%) over normal operating times. The humidity measurement is factored into the air density calculation to increase accuracy when recording airspeed (electronic supplementary material, SM.2).

*Smoke purge valve*. To allow smoke visualization in the test section, a smoke purge valve was included in the ceiling of the diffuser downstream of the fan. The motorized valve is operated through the Human Machine Interface. Smoke is driven out by the fan pressure while fresh air is pulled in through the breather gap.

## 3. Tunnel performance

Following installation, tests were performed to quantify the aeroacoustic properties of the wind tunnel. The flow in the test section is stable (1*σ* airspeed = 0.018 m s^−1^ at the mid-range speed, 25 m s^−1^), uniform (1*σ* airspeed = 0.023 m s^−1^ at 25 m s^−1^), straight (1*σ* deviation angle = 0.14° at 25 m s^−1^), low turbulence (axial turbulence intensity = 0.021% at 25 m s^−1^) and quiet (81.8 dB at 25 m s^−1^). The datasets resulting from the aeroacoustic testing have been uploaded as part of the electronic supplementary material. Details of the testing procedures and results are given below.

### 3.1. Aeroacoustic testing traverse

A custom traverse was designed to measure the flow properties in the test section (electronic supplementary material, figure SF.2). The base of the traverse was a steel strut positioned at one of four horizontal positions (200 mm spacing) and four vertical positions (160 mm spacing) at the back end of the test section. A steel sting attached to the strut extended upstream to a point approximately 1 m downstream of the test section entrance. The result was a 4 × 4 grid with 16 positions at which airspeed, pressure, flow angularity, temperature and turbulence were measured. The strut can also be positioned such that measurements are taken at the centreline of the test section. For each type of measurement, a different probe was mounted on the end of the sting: for airspeed and pressure measurements, a Pitot-static probe (Kanomax 1 m; sent to a MKS 690A/698A transducer); for flow angularity, a flow angle probe (CEA 5-hole; sent to a MKS 690A/698A transducer); for turbulence intensity, a hotwire probe (TSI X-Film Model 1241–20 with a CTA Thermal Anemometer AN-1003 system); and for temperature, a resistance thermometer. All measurements were conducted at a stable tunnel temperature of 20°C.

### 3.2. Airspeed stability and uniformity

**Figure 2.**
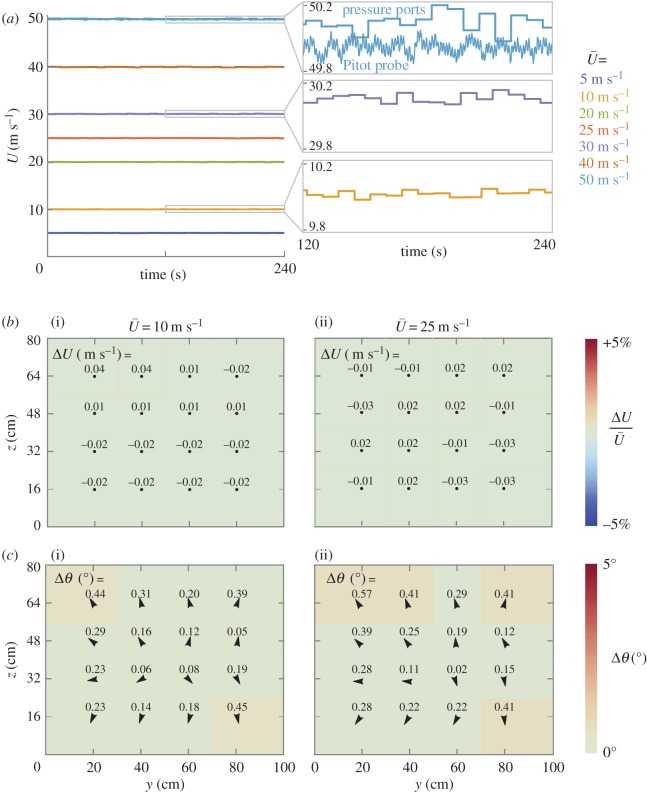
Flow in the test section remains stable over the operating range and uniform in both magnitude and direction. (*a*) A range of airspeeds, *U*, measured by pressure ports in the stilling chamber and contraction are shown while the fan operates at fixed speeds (r.p.m.). The zoomed inserts show how instantaneous airspeed differs slightly from the mean airspeed, $\bar{U}$. The standard deviation of *U* ranges from 0.017 m s^−1^ when $\bar{U}$ = 10 to 0.060 m s^−1^ when $\bar{U}$ = 50 m s^−1^. The maximum speed (50 m s^−1^) was checked with additional airspeed data from a Pitot-static probe at the test section centreline. (*b*) The deviation in airspeed from the average airspeed, Δ*U*, is measured on a 4 × 4 grid in the test section (black dots indicate grid points). Deviations are comparable between the 10 m s^−1^ airspeed case ((i) 1*σ* of Δ*U* = 0.020 m s^−1^) and the 25 m s^−1^ airspeed case ((ii) 1*σ* of Δ*U* = 0.023 m s^−1^). Colour indicates the relative airspeed deviation, that is, $\Delta U/\bar{U}$. (*c*) The angle between the flow vector and a streamwise unit vector, Δ*θ*, is measured on a 4 × 4 grid in the test section (black arrows indicate grid points). The arrows show the direction of the flow velocity projected into the plane of the grid (*z*–*y*-plane). The flow shows a slight tendency to diverge at the plane of the grid but is primarily straight for both the 10 m s^−1^ airspeed case ((i) 1*σ* of Δ*θ* = 0.13°) and the 25 m s^−1^ airspeed case ((ii) 1*σ* of Δ*θ* = 0.14°).

The airspeed in the test section remained stable over the full operating range (0–50 m s^−1^; figure 2*a*). To test stability, airspeed was recorded for 240 s at 1 Hz from the calibrated pressure ports. Seven set point airspeeds were considered over the operating range: 5, 10, 20, 25, 30, 40 and 50 m s^−1^. Airspeed was more steady at the lowest speed (1*σ* = 0.017 m s^−1^) than the highest speed (1*σ* = 0.060 m s^−1^), but all airspeeds remained stable to within less than 0.2% of the mean. The highest airspeed value (50 m s^−1^) was confirmed using measurements from the Pitot-static probe at the centreline.

The airspeed was spatially uniform in the test section (figure 2*b*). To test uniformity, airspeed was recorded from the Pitot-static probe (60 s at 10 Hz) at each position of the 4 × 4 testing grid. Airspeed uniformity was comparable at the two airspeeds considered: 1*σ* = 0.020 m s^−1^ at 10 m s^−1^ and 1*σ* = 0.023 m s^−1^ at 25 m s^−1^. For completeness, we also present the total pressure uniformity by using data from only the front port of the Pitot-static probe (electronic supplementary material, figure SF.3). Total pressure variation was slightly lower at 10 m s^−1^ (1*σ* = 0.344 Pa) compared with 25 m s^−1^ (1*σ* = 0.970 Pa). The uniformity in both airspeed and total pressure demonstrate that total pressure, static pressure and dynamic pressure are all uniform in the test section over the full operating range.

The direction of the flow was straight and uniform in the test section (figure 2*c*). A flow angle probe measured flow direction by comparing pressure readings from two ports on opposing sides of the probe. The probe was calibrated by performing a roll axis angle sweep in both upright and inverted positions. Flow angle was then recorded from the probe (60 s at 10 Hz) at each position of the 4 × 4 testing grid. Yaw and pitch flow angles were measured separately, and then the total angle was calculated between flow velocity and a streamwise unit vector (electronic supplementary material, SM.3). This total angle showed little variation over the test section at each airspeed considered: 1*σ* = 0.13° at 10 m s^−1^ and 1*σ* = 0.14° at 25 m s^−1^. The low levels of flow angularity are made possible by the turning vanes and flow straightening elements upstream.

### 3.3. Temperature and humidity stability and uniformity

**Figure 3.**
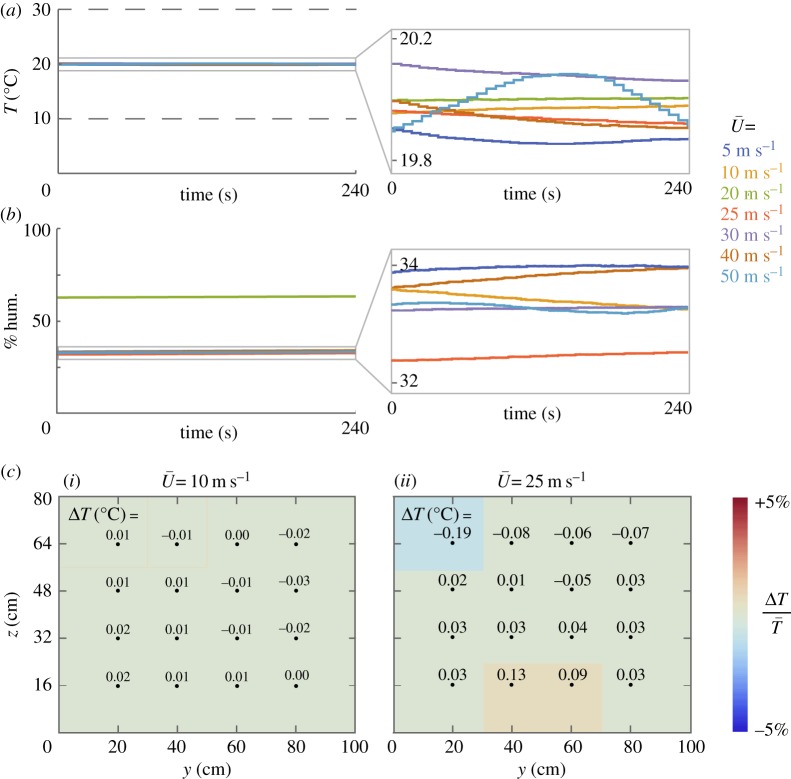
Temperature and humidity remain stable over the operating range; temperature is uniform in the test section. (*a*) Temperature in the stilling chamber is shown while a proportional-integral controller regulates a water-chilled heat exchanger to keep the tunnel at 20°C. Dashed lines show the upper and lower limits of the tunnel's operating range. The zoomed insert shows that temperature deviations from the mean temperature, $\bar{T}$, vary depending on average airspeed, $\bar{U}$. The standard deviation of *T* ranges from 0.007°C when $\bar{U}$ = 10 m s^−1^ to 0.073°C when $\bar{U}$ = 50 m s^−1^. (*b*) Relative humidity is recorded during tunnel operation. One trial ($\bar{U}$ = 20 m s^−1^) was conducted on a different day, resulting in a different average humidity (approx. 63%) than the other trials (approx. 32–34%). The zoomed insert shows that relative humidity changes only slightly with no clear dependence on airspeed; the average standard deviation across the seven airspeeds tested was 0.08%. (*c*) The deviation in temperature from the average tunnel temperature, Δ*T*, is measured on a 4 × 4 grid in the test section (black dots indicate grid points). Deviations are slightly lower when $\bar{U}$ = 10 m s^−1^ ((i) 1*σ* of Δ*T* = 0.015°C) compared with when $\bar{U}$ = 25 m s^−1^ ((ii) 1*σ* of Δ*T* = 0.074°C). Colour indicates the relative temperature deviation, that is, $\Delta T/\bar{T}$.

The temperature and relative humidity remained stable over the full operating range (0–50 m s^−1^; figure 3*a*). To test stability, temperature and humidity were recorded by the facility probes in the stilling chamber (240 s at 1 Hz). Seven set point airspeeds were considered over the operating range: 5, 10, 20, 25, 30, 40 and 50 m s^−1^. Temperature was more steady at the lowest speed (1*σ* = 0.007°C) than the highest speed (1*σ* = 0.073°C), but all temperatures remained stable to within less than 0.4% of the mean. The stability of temperature demonstrates the effectiveness of the manually tuned proportional-integral controller and the constant flow heat exchanger. As relative humidity was not actively controlled, its value is primarily a function of the ambient laboratory conditions. For example, the 20 m s^−1^ condition was measured on a different day than the other airspeeds, resulting in the different humidity value recorded for that airspeed.

The temperature was also spatially uniform in the test section (figure 3*b*). To test uniformity, temperature was recorded by a resistance thermometer (60 s at 10 Hz) at each position of the 4 × 4 testing grid. The facility temperature changes slightly in the time it takes to switch between grid point trials. To separate the temporal and spatial variations in temperature, temperatures were corrected by comparing to an average facility temperature (electronic supplementary material, SM.4). Temperature was uniform at each of the two flow airspeeds considered, though more uniform at the lower of the two airspeeds: 1*σ* = 0.015°C at 10 m s^−1^ and 1*σ* = 0.074°C at 25 m s^−1^. The high levels of uniformity demonstrate the effectiveness of the streamlined fins in the constant flow heat exchanger.

### 3.4. Turbulence intensity

**Figure 4.**
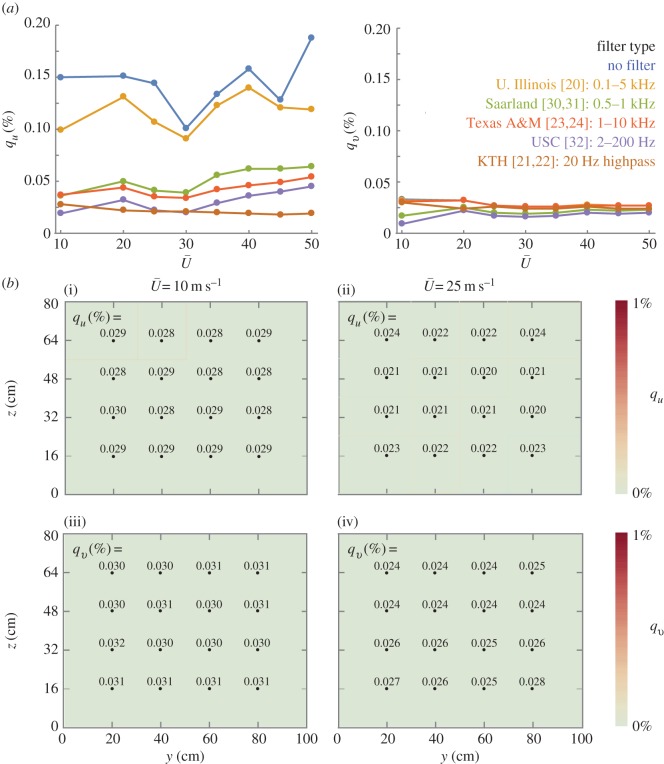
Axial and transverse turbulence intensities stay low throughout the test section over the full operating range. (*a*) Turbulence intensity in the Stanford wind tunnel is plotted unfiltered and with filter types from other wind tunnel publications. The axial and transverse turbulence intensities, *q_u_* and *q_v_*, are the ratios of root mean square fluctuations in streamwise and vertical airspeeds to the average airspeed: $\bar{u\prime }/\bar{U}$ and $\bar{v\prime }/\bar{U}$, respectively. Axial turbulence intensity shows a strong dependence on the cutoff frequency of a highpass filter. All conditions were measured at the centreline, except for 10 m s^−1^ which is the average of the four central points of the grid. The filters are chosen to facilitate comparison with existing wind tunnel publications, where 0.1–5 kHz bandpass (U. Illinois [20]), 0.5–1 kHz bandpass (Saarland [7,8]), 1 Hz–10 kHz bandpass (Texas A&M [23,24]), 2–200 Hz bandpass (USC [30]), and 20 Hz highpass (KTH [21,22]) filters have been used to report intensity. Note: because our sampling rate was 10 kHz, any cutoff frequencies above 5 kHz had no effect on our reported intensities. Transverse turbulence intensities show much less dependence on cutoff frequency, presumably because of low-frequency streamwise travelling waves. Data from the curves are tabulated in electronic supplementary material, tables ST.1 and ST.2. (*b*) Axial ((i)(ii)) and transverse ((iii)(iv)) turbulence intensities are measured over a 4 × 4 grid in the test section (black dots indicate grid points). A 20 Hz highpass filter was applied to the data following the methods used to quantify the KTH tunnel [14,15]. The intensities are uniform, both for the 10 m s^−1^ airspeed case ((i)(iii) 1*σ* of *q_u_* = 0.001%; 1*σ* of *q_v_* = 0.001%) and the 25 m s^−1^ airspeed case ((ii)(iv) 1*σ* of *q_u_* = 0.001%; 1*σ* of *q_v_* = 0.006%).

Turbulence intensities are low in the test section over the full operating range (0–50 m s^−1^; figure 4*a*). Two turbulence intensities were considered: the axial turbulence intensity, $q_{u}\equiv \bar{u\prime }/\bar{U}$, and the transverse turbulence intensity, $q_{v}\equiv \bar{v\prime }/\bar{U},$where $\bar{u\prime }$ and $\bar{v\prime }$ are the root mean square fluctuations in streamwise and vertical flow speed, respectively, and $\bar{U}$ is the time-averaged bulk flow speed. To calculate turbulence intensities, velocity data were sampled at 10 kHz using an x-film hot wire probe (TSI Model 1241–20). The frequency response rate of the probe is airspeed dependent, but it is always above 100 kHz and thus more than 10 times our sampling rate. The AC and DC components of the signal were sampled together (DC coupled), with no gain or offset, using a low-noise anemometer (CTA Thermal Anemometer AN-1003). The maximum signal to noise ratio for the sampled signal was 96 dB based on the 16 bit analogue-to-digital conversion. Notch filters were applied between 60*i* ± 1 Hz, where *i* = 1, 2, 3, … , to remove electrical noise from the velocity signal (electronic supplementary material, figure SF.4). The energy content of the turbulence was concentrated below 1 kHz (electronic supplementary material, figure SF.5). At all airspeeds considered (10, 20, 25, 30, 35, 40, 45 and 50 m s^−1^), both axial and transverse turbulence at the centreline were found to be low compared with existing animal flight and small flight vehicle wind tunnels: *q_u_* ≤ 0.028% and *q_v_* ≤ 0.030%.

Highpass filtering has a large effect on the axial turbulence intensities reported [15]. Previous studies have used varying filters, documented with varying degrees of precision, making it difficult to compare the turbulence performance of existing tunnels. To overcome this complication, we applied five different filter types based on the entries of table 1 (see electronic supplementary material, SM.5 for detail on the filtering and analysis technique). Filtering has only a moderate effect on transverse turbulence intensities, but a significant effect on axial turbulence intensities. This is to be expected since low-frequency streamwise travelling waves primarily affect axial flow speeds. A comparison of unfiltered data suggests that our facility has a higher travelling wave contribution than the KTH wind tunnel (average central *q_u_* = 0.144% at 10 m s^–1^ compared with less than 0.1% reported at KTH at 10 m s^−1^) [14].

Turbulence intensities were not only low at the centreline, but also throughout the test section (figure 4*b*). Axial and transverse turbulence intensities were measured at each grid point using the same technique as was used at the centreline (TSI X-Wire Model 1241–20). Both axial and transverse intensities showed little variation across the 4 × 4 grid (1*σ* < 0.01%) for both speeds at which the uniformity was measured.

### 3.5. Acoustics

A separate mount was built for the acoustic probe (electronic supplementary material, figure SF.6). A forward-swept fibreglass strut extended through a slotted floor panel in the bottom of the test section. The strut was supported by an aluminium frame mounted to the bottom of the test section. Acoustic levels were measured at the end of the strut using a 60 kHz microphone (B&K 0.5 in 4166). To improve accuracy, the signal was sent through a signal conditioner (B&K NEXUS 4-Channel) and a pre-amplifier (B&K 2669), resulting in a hardware-induced 60 Hz highpass filter. The cooling pump was on during acoustic testing, and tape was placed over screw hole indentations in the frame, as well as gaps between movable windows and the test section, as is common practice for acoustic measurements.

**Figure 5.**
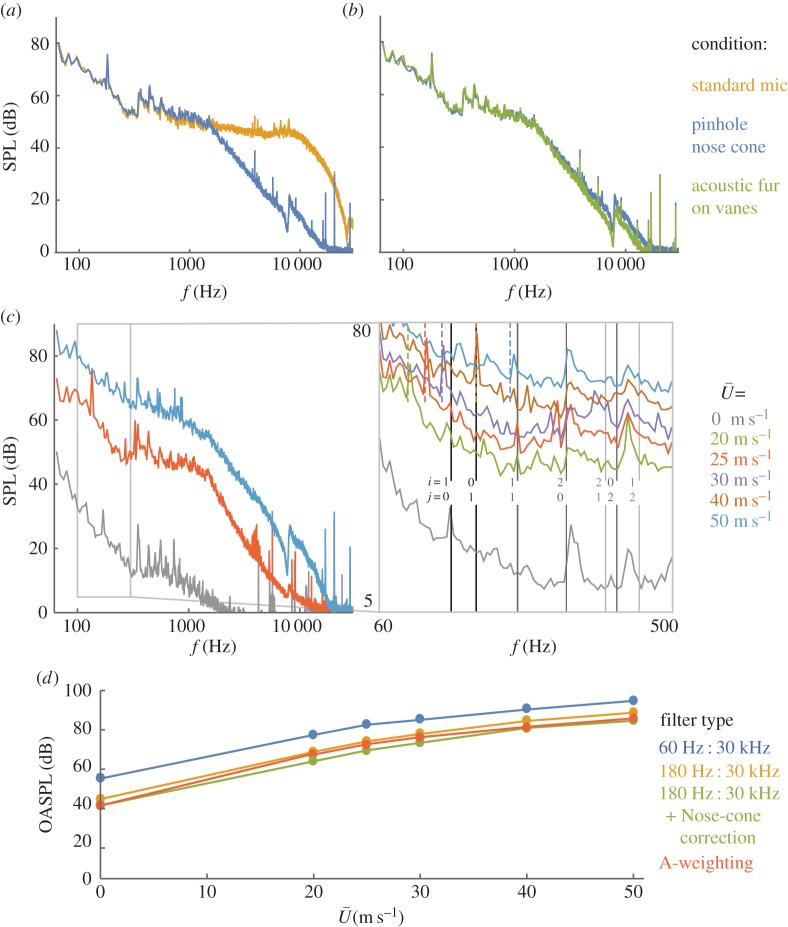
Noise levels in the test section are barely audible at low speeds, then increase with airspeed; noise decreases steadily with frequency except for localized tones. (*a*) SPL is measured at the centreline of the test section, both with a standard nose cone (standard mic) and with a GRAS custom pinhole nose cone mounted on the front. The nose cone was used to attenuate high-frequency turbulence (above 1500 Hz), but it also caused a slight amplification of frequencies below 1500 Hz and a slight jump in SPL around 8000 Hz, presumably due to resonance in the nose-cone cavity. All further trials were performed with the nose cone attached. (*b*) Adding pile fabric to the leading edge of the acoustic turning vanes in corner 1 causes a slight decrease in SPL, especially at high frequencies. (*c*) SPLs decrease with increasing frequency and have localized tones. The zoomed insert shows the tones more clearly, along with expected test section resonant frequencies: the blade passing frequency (coloured dashed lines, 18 blades × fan frequency = 107, 133, 158, 209, 260 Hz) and the acoustic harmonics based on width and height of the test section (grey solid lines; independent of airspeed; *i* and *j* are the numbers of horizontal and vertical standing waves, respectively, in the acoustic harmonic mode). (*d*) Overall SPLs increase with airspeed, ranging from 53.2 dB at 0 m s^–1^ to 94.5 dB at 50 m s^−1^ with no filtering beyond the built-in 60 Hz highpass filter. A 180 Hz filter, a nose-cone transfer function, and an A-weighting filter are used to demonstrate the effect of highpass filtering and resonance correction (electronic supplementary material, table ST.3).

Following Johansson [22], a streamlined GRAS custom pinhole nose cone was attached to the front of the microphone before measuring acoustic levels. The main effect of the nose cone is to attenuate the sound pressure level (SPL) at frequencies above 1500 Hz (figure 5*a*), which are presumed to be a result of small-scale turbulence and therefore not relevant to the acoustic signature of the wind tunnel [22]. The nose cone also introduced a small jump in SPL around 8 kHz, presumably due to resonance in the nose cone. This effect is negligible, because the SPL contribution at these high frequencies (approx. 10 dB) is several orders of magnitude lower than contributions in the 60–1500 Hz range (approx. 70 dB). However, at some frequencies below 1500 Hz, nose-cone resonance increased the acoustic response by up to 8 dB. This amplification is likely to cause the reported overall acoustic levels to be slightly overestimated.

To reduce noise further, pile fabric (synthetic fur) was wrapped around the leading edge of the acoustic turning vanes in corner 1 (electronic supplementary material, figure SF.7). This addition of pile fabric led to a small but measurable decrease in the test section SPL, especially at higher frequencies (figure 5*b*).

Frequency contributions to sound levels in the test section showed a slow decrease in magnitude with several localized tones (figure 5*a–c*). Many of the tones occur in the zero airspeed condition and are therefore attributed to acoustic eigenmodes in the test section. The *i*-*j*th transverse eigenfrequency, *f_ij_*, of a rectangular air column is *f_ij_* = (*c*/2)[(*i*/*w*) + (*j*/*h*)^2^]^1/2^ [35], where *c* is the speed of sound and *w* and *h* are the width and height of the air column, respectively. For the test section, the first few eigenfrequencies occur in the 100–500 Hz range (figure 5*c*), where many of the local SPL spikes occur. The spikes are not expected to line up perfectly with the eigenfrequencies, because the tunnel walls diverge. Still, the first two horizontal modes (*i* = 1, 2; *j* = 0) show local spikes in SPL close to their predicted values. Other spikes below 250 Hz can be attributed to the blade passing frequency, which causes a small airspeed-dependent spike in the SPL.

Overall, acoustic levels in the test section are low; at the lowest airspeeds the wind tunnel is barely audible above the room background noise. The overall sound pressure level (OASPL) rises steadily with airspeed, with unfiltered values ranging from 53.2 dB at 0 m s^−1^ to 76.4 dB at 20 m s^−1^ to 94.5 dB at 50 m s^−1^ (figure 5*d*). As with turbulence intensity, the frequency cutoff of a highpass filter has a significant effect on the reported OASPL, making it difficult to compare existing wind tunnels. For this reason, we report our results with multiple filters. The first filter is the hardware-induced 60 Hz highpass filter from the signal conditioner. A 180 Hz filter was applied with the same routine that was used for turbulence intensity (electronic supplementary material, SM.5). For a third filtering option, we demonstrate the effect of applying a transfer function inspired by Johansson [22] to account for nose-cone amplification below 1500 Hz (electronic supplementary material, table ST.3). Finally, we apply A-weighting (electronic supplementary material, table ST.3) to estimate noise levels based on human perception of sound.

### 3.6. Active turbulence grid

**Figure 6.**
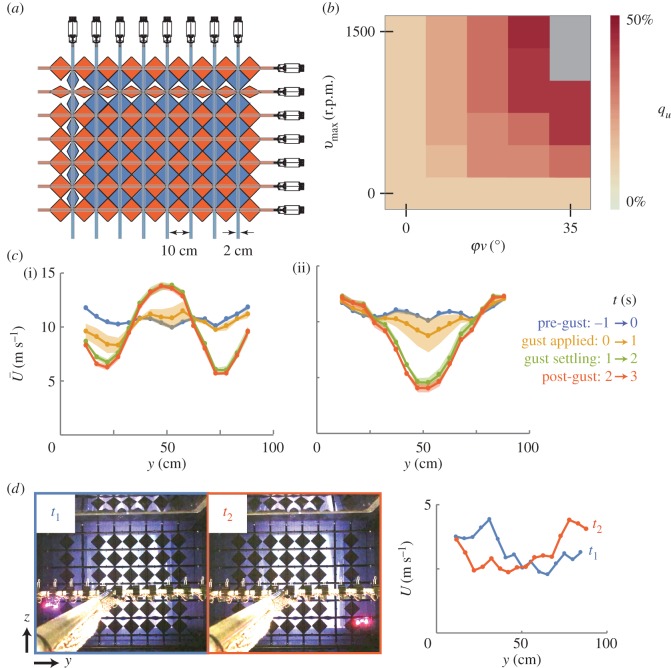
An active turbulence grid can be used to create turbulence or non-uniform flows in the test section. (*a*) A schematic of the active turbulence grid shows how 8 vertical and 7 horizontal vanes can be independently rotated to perturb the flow upstream of the test section. (*b*) Grid motions based on those by Cekli *et al*. [33,34] produce axial turbulence intensities ranging from approximately 12.5% to approximately 45.1% (based on unfiltered velocity data). For these motions, the vertical vanes were held at a fixed angle while the horizontal vanes oscillated with a prescribed amplitude, *φ*_H_, and max rotation speed, *v*_max_. Grey boxes correspond to *φ*_H_–*v*_max_ conditions beyond the physical limitations of the motors. These data are shown in tabular form in electronic supplementary material, table ST.4. (*c*) Grid vanes were switched between fixed positions to change a uniform flow to a jet or wake flow (electronic supplementary material, figure SF.8). The average airspeed at 16 *y* positions shows a jet (i) and wake (ii) after around 1 s. The curves and shaded bands show an average and standard deviation of three identical trials. For each trial, airspeeds were averaged over 1 second before the vanes switched positions (−1 s < time(*t*) ≤ 0 s) and each of the 3 s after the vanes switched positions (0 s < *t* ≤ 1 s; 1 s < *t* ≤ 2 s; 2 s < *t* ≤ 3 s). (*d*) Vane positions can adapt based on the position of a tracked animal or vehicle. Here, the concept is demonstrated by opening one vertical vane upstream of a small quadcopter with a marker tracked by a motion capture system. Instantaneous airspeed profiles are shown at two time steps (*t*_1_ and *t*_2_) corresponding with two frames from the video (electronic supplementary material, video SV.1; selected frames processed with Adobe Photoshop: Max Levels to 80 to improve contrast).

The active turbulence grid can increase turbulence intensities up to 16 or 45%, depending on the highpass filter used. To facilitate comparison with other studies using active grids, we applied a grid motion that has been studied extensively [33,34]. For this motion, the vertical vanes stay at a fixed angle to generate constant blockage, and the horizontal vanes oscillate with a given maximum velocity, *v*_max_, and amplitude, *φ*_H_. We studied the axial turbulence intensity, *q_u_*, over a range of motion inputs spanning the operating range of the motors driving the vanes (5 × 6 grid equally spaced over the domain [*v*_max_, *φ*_H_] ∈ [0, 1500] r.p.m. × [0, 35]°). For each motion, 120 s of data were sampled at 10 kHz from a single hotwire probe (Dantec 55P16; CTA module Dantec 54T42) placed on the testing sting at the centreline of the test section. Unfiltered axial turbulence intensities ranged from 12.5% ([*v*_max_, *φ*_H_] = [0, 0]) to 45.1% ([*v*_max_, *φ*_H_] = [1200 r.p.m., 26.25°]) (figure 6*a*). The low value occurs when the vanes are fully open and motionless, passively introducing turbulence into the flow. As with the intensities in the empty test section, applying a highpass filter changes the reported intensity. With a 0.1 Hz highpass filter, the range changes to 12.4–44.6%; with a 1 Hz highpass filter, 12.0–40.1%; with a 20 Hz highpass filter, 8.1–16.1%. The significant reduction in maximum turbulence intensity with the 20 Hz highpass filter demonstrates the fact that the grid is primarily injecting turbulence at low frequencies, which do not have time to decay into broadband turbulence since the flow is not likely to be fully developed by the time it reaches the probe [36]. We chose to focus on unfiltered turbulence values for the active grid, because unlike the flow with no grid, where low-frequency perturbations represent travelling waves in the tunnel, the flow with the grid has low-frequency perturbations that have been injected purposefully.

The active grid can also be used to create non-uniform velocity profiles that include values up to ±40% of the mean airspeed. These profiles are created by adjusting the fixed position of the vanes. Changing the vane position can change the blockage and therefore the pressure drop across the active grid, which changes the mean airspeed in the tunnel at a fixed fan speed. When the airspeed changes, it can take approximately 10 s to reach a new equilibrium as the mass of air is accelerated in the tunnel. One way to avoid this delay is by switching between profiles with the same mean airspeed. We used this concept to demonstrate a switch from uniform flow to jet and wake profiles in approximately 1 s (figure 6*b*). The profiles were measured with an array of 16 wind sensors (Modern Device Rev P hot film; 10 Hz [37]) spaced 5 cm apart horizontally, mounted on the testing sting centred in the test section. Airspeed data for each sensor were collected at 10 Hz and averaged over 30 s.

While the 1 s delay is still longer than the timescales of wingbeats or rapid flight manoeuvres, the profiles can adapt to the changing position of an animal or vehicle. We demonstrated this possibility by adding a 4 mm diameter retroreflective marker to the base of a small quadcopter (Estes Proto X) flying downstream of the active grid. Six infrared cameras (Qualisys Oqus 7 plus; 1000 Hz; 3 MP) tracked the position of the quadcopter, and the position data were relayed to the active grid control system. All horizontal vanes were held fully open, and all vertical vanes were closed except for the one closest to the lateral position of the quadcopter. The result is a jet with constant blockage that follows the position of the quadcopter (figure 6*c*). While the quadcopter was moving too quickly for the jet to match its position in real time, the successful tracking shows that the active grid could send gust perturbations using a closed-loop controller based on the positions/orientations of animals/vehicles in the test section.

## 4. Conclusion

The aeroacoustic performance of the new wind tunnel at Stanford is comparable to existing state-of-the-art wind tunnels. We obtained streamwise turbulence levels less than or equal to 0.030% throughout the test section at 10 m s^−1^, compared with less than 0.04% at KTH [21] using the same filtering technique, demonstrating comparable turbulence levels. While we are unable to recreate the filtering used at Lund [27–29], their use of 1024 samples taken at 1 kHz suggests an effective highpass cutoff frequency of approximately 1 Hz. When using a 1 Hz highpass frequency, our streamwise turbulence intensities were approximately 0.05%, comparable to the values reported at Lund. These values are also comparable to those at Texas A&M, where 1 Hz was also used as a lower cutoff frequency [23,24]. The wind tunnel at KTH used similar acoustic treatment and testing, allowing a straightforward comparison of our sound levels. Noise levels of 69 dB were reported at 35 m s^−1^ in the KTH tunnel, which is lower than our reported 84.4 dB [22]. The difference with KTH could be somewhat exaggerated because of the transfer function used during acoustic measurements at KTH. When we apply a transfer function inspired by theirs, our reported levels decrease by approximately 6% (electronic supplementary material, table ST.3). Even with this filter the KTH tunnel is more quiet in comparison; regardless, both tunnels are barely audible in the test section at animal flight airspeeds.

Our new wind tunnel has comparatively low turbulence, low flow angularity, high flow uniformity and high temperature uniformity. Two additional features make our animal flight wind tunnel unique: the ability to switch from open to closed jet configuration and the ability to tune turbulence intensity with an active grid. Existing tunnels are uniquely suited for studying different aspects of animal flight. With the ability to vary climb angle, the tunnel at Lund [27,29] is ideal for studying migration and ecology. With the ability to vary pressure, the tunnel at Western Ontario [25,26] is ideal for studying high-altitude flight and bioclimatology. The abilities to vary turbulence and perform open-jet experiments with biplanar fluoroscopy make the new wind tunnel at Stanford ideal for studying comparative biomechanics.

## Supplementary Material

Supplemental Materials, Figures, and Tables; Raw wind tunnel data

## Data accessibility

Data available from the Dryad Digital Repository: http://dx.doi.org/10.5061/dryad.b65fj [38].
