# Supplementary material for: A new low-turbulence wind tunnel for animal and small vehicle flight experiments
Source: R Soc Open Sci. 2017 Mar 29;4(3):160960. doi: 10.1098/rsos.160960 (PMC5383841; doi:10.1098/rsos.160960)
Supplement: Supplemental Materials, Figures, and Tables; Raw wind tunnel data [file rsos160960supp1.pdf]

Supplemental Material SM.1
The divergence angles of the test section walls affect pressure gradients

The floor and ceiling divergence angle is fixed (0.5°) while the sidewall divergence angle is variable to adjust for different blockage levels. For example, a numerical boundary layer solver was applied to the contraction and test section at a testing speed of 10 m/s. The solver estimated a 3.7 mm displacement thickness at the test section entrance (δ_{ent}) and a 7.1 mm displacement thickness at the exit (δ_{exit}). With an empty test section, the change in pressure Δp across the test section can be estimated as

$$\Delta p = \left[1 - \left(\frac{A_{\mathrm{ent}}}{A_{\mathrm{exit}}}\right)^2\right]\frac{1}{2}\rho U^2$$

where ρ is the air density, U is the airspeed, and A_{ent} and A_{exit} are the effective areas at the test section entrance and exit. The effective areas can be estimated using the displacement thicknesses at the entrance and exit:

$$A_{\mathrm{ent}} = (w - 2\delta_{\mathrm{ent}})(h - 2\delta_{\mathrm{ent}})$$
$$A_{\mathrm{exit}} = (w - 2\delta_{\mathrm{exit}} + 2l\theta_{\mathrm{side}})(h - 2\delta_{\mathrm{exit}} + 2l\theta_{\mathrm{top}}),$$

where w, l, and h are the width, length, and height of the tunnel, respectively; and θ_{side} and θ_{top} are the divergence angles of the side and top walls, respectively. Since θ_{top} is fixed to 0.5°, θ_{side} can be tuned to minimize Δp. In the case of the empty test section, Δp is minimized with $\theta_{\mathrm{side}} = -0.37°$, that is, side walls rotated inward from parallel by 0.37 degrees. This analysis could be repeated and confirmed experimentally to minimize pressure gradients under conditions in which the test section is not empty.

Supplemental Material SM.2
The airspeed in the test section is calculated using pressure, temperature, and humidity readings

The pressure ports installed in the tunnel give two readings: (1) a differential pressure, Δp, between the stilling chamber and the end of the contraction, and (2) a gauge pressure, p_{G}, at the end of the contraction by comparing static pressure to atmospheric pressure. To calculate airspeed in the test section, two different values of pressure are needed: the stagnation pressure in the test section, p_0, and the static pressure in the test section, p. During commissioning, a pitot-static probe was used to create an empirical second-order polynomial mapping from Δp and p_{G} to p_0 and p:

$$p_0(\Delta p, p_{\mathrm{G}}) = p_{\mathrm{G}} + \alpha_0 + \alpha_1\Delta p + \alpha_2(\Delta p)^2,$$

$$p(\Delta p, p_G) = p_G + \beta_0 + \beta_1 \Delta p + \beta_2 (\Delta p)^2,$$

where α_i and β_i are empirically fitted coefficients. With the stagnation and static pressure in the test section known, the airspeed follows from equations for isentropic flow of an ideal gas [1]. First, the Mach number, M, in the test section can be calculated from the ratio of stagnation to static pressure:

$$M = \sqrt{\frac{2}{\gamma - 1}\left[\left(\frac{p_0}{p}\right)^{\frac{\gamma-1}{\gamma}} - 1\right]},$$

where γ is the ratio of specific heats for air ($\gamma = 1.4$). The static temperature in the test section, T, is calculated from the stagnation temperature measured in the stilling chamber, T_0:

$$T = \frac{T_0}{\left(1 + \frac{\gamma - 1}{2} M^2\right)},$$

To account for humidity, the specific gas constant is estimated for the air and water vapor mixture. First, the equilibrium vapor pressure for water, $p_{\mathrm{H20,Eq}}$, is calculated from an empirical formula [6],

$$p_{\mathrm{H20,Eq}} = 6.11 * 10^{\frac{7.5\,T}{237.3+T}},$$

where $p_{\mathrm{H20,Eq}}$ is in Pascals and T is in degrees Celsius. The partial pressure of water, p_{H20}, follows from the definition of relative humidity,

$$p_{\mathrm{H20}} = (\%\ \mathrm{humidity}) * p_{\mathrm{H20,Eq}}.$$

The specific gas constant for the air-water mixture, R, can now be calculated by using a weighted fraction of the molecular weights of air, M_{air}, and water, M_{water}:

$$R = \frac{\tilde{R}}{\frac{p_{\mathrm{H20}}}{p} M_{\mathrm{air}} + (1 - \frac{p_{\mathrm{H20}}}{p}) M_{\mathrm{water}}},$$

where $\tilde{R}$ is the universal gas constant ($R \approx 8.314$ J mol^{-1}K^{-1}). With the modified gas constant, the speed of sound of the air-water mixture, a, can be calculated using

$$a = \sqrt{\gamma R T}.$$

The airspeed in the test section, U, now follows from the definition of Mach number,

$$U = Ma.$$

Supplemental Material SM.3
The total angle of the flow velocity can be calculated from yaw and pitch values

The total angle, θ, between a unit vector in the flow direction, $\hat{\boldsymbol{u}} \equiv <\hat{u}, \hat{v}, \hat{w}>$, and a unit vector in the streamwise direction, $\widehat{\boldsymbol{e}_x}$, can be determined using the definition of the dot product:

$$\theta = \cos^{-1}(\hat{\boldsymbol{u}} \cdot \widehat{\boldsymbol{e}_x}) = \cos^{-1}(\hat{u}).$$

We can write $\hat{u}$ in terms of the measured yaw and pitch angles (θ_{yaw} and θ_{pitch}) by noting that

$$\tan(\theta_{\text{yaw}}) = \frac{\hat{v}}{\hat{u}} \text{ and } \tan\left(\theta_{\text{pitch}}\right) = \frac{\hat{w}}{\hat{u}},$$

and thus

$$\tan(\theta_{\text{yaw}}) = \frac{\sqrt{1 - \hat{u}^2 - \hat{w}^2}}{\hat{u}} = \frac{\sqrt{1 - \hat{u}^2 - \hat{u}^2 \tan^2(\theta_{\text{pitch}})}}{\hat{u}}.$$

Solving for u gives

$$\hat{u} = \frac{1}{\sqrt{1 + \tan^2\left(\theta_{\text{pitch}}\right) + \tan^2\left(\theta_{\text{yaw}}\right)}}.$$

Thus, the total angle θ between the flow and the streamwise direction can be written as

$$\theta = \cos^{-1}\left(\frac{1}{\sqrt{1 + \tan^2\left(\theta_{\text{pitch}}\right) + \tan^2\left(\theta_{\text{yaw}}\right)}}\right).$$

Supplemental Material SM.4
Temperature uniformity measurements were corrected for temporal variations in facility temperature

The facility temperature has small temporal variations with similar magnitude to the spatial temperature variations in the test section. For this reason, a special procedure is applied to separate the temporal and spatial effects of temperature variation.

First, a ratio of total temperatures is taken between the grid point measurement, T', and the facility measurement in the stilling chamber, T_{SC}. This ratio is assumed to be a function of test section position and not facility temperature. These ratios are then multiplied by the average facility temperature measured over all grid point trials, $\overline{T_{\mathrm{SC}}}$, to give the corrected grid point temperature, T:

$$T = \frac{T'}{T_{\mathrm{SC}}}\overline{T_{\mathrm{SC}}},$$

where all temperature values are written in degrees Kelvin. The corrected variations in ΔT are the corrected temperatures minus the average of all corrected temperatures over the grid, $\bar{T}$:

$$\Delta T = T - \bar{T}.$$

These corrected ΔT values are reported in figure 3b in the main manuscript. For completeness, we present the uncorrected temperature uniformity data, that is, $\Delta T' = T' - \bar{T}'$, in supplemental figure SF.8, where it can be seen that the correction has a small but noticeable effect on the reported temperature uniformity, particularly at the lower speed (10 m/s).

Supplemental Material SM.5
Turbulence intensities are reported with three different highpass filters

Reported turbulence intensities are affected by low frequency traveling waves in the tunnel, and it is therefore customary to explore the effect of a highpass frequency cutoff when reporting turbulence intensity [2]. We therefore applied five bandpass or highpass filters to our velocity data that are representative for previous studies. Since previous studies have used different definitions of the Discrete Fourier Transform, we provide here a detailed description of our filtering technique, which is the same as the technique used by Lindgren [3] (see Lindgren [3] Paper 3: Equations 19, 20, 21; note: author uses zero-indexing so his indices differ by 1).

The first step of our filtering technique is to define axial and transverse fluctuation speeds:

$$u' \equiv U - \bar{U}$$
$$v' \equiv V - \bar{V},$$

where U and V are the streamwise and vertical components of flow velocity, and overbars denote time averages. Since velocity data were taken at 10 kHz for 60 seconds, u' and v' are vectors with length n = 600,000. We then define the axial and transverse Discrete Fourier Transforms of the scaled fluctuation speeds:

$$\mathcal{F}_u \equiv \frac{1}{n}\sum_{j=1}^{n} \frac{u'_j}{\bar{U}} e^{\frac{2\pi i(j-1)(i-1)}{n}}, i = 1, 2, 3 \dots n$$
$$\mathcal{F}_v \equiv \frac{1}{n}\sum_{j=1}^{n} \frac{v'_j}{\bar{U}} e^{\frac{2\pi i(j-1)(i-1)}{n}}, i = 1, 2, 3 \dots n,$$

where u'_j and v'_j are the j'th elements in u' and v'. Like the fluctuation speeds, the Discrete Fourier Transforms $\mathcal{F}_u$ and $\mathcal{F}_v$ are vectors of length n. The vector of frequencies, f, corresponding to each element of the Discrete Fourier Transform is

$$f = \frac{i-1}{60}\ \mathrm{Hz},\quad i = \ 1, 2 \ldots\ n.$$

For example, the i'th element of $\mathcal{F}_u$ represents the contribution of the i'th element of f to the axial velocity signal.

The axial and transverse turbulence intensities can be calculated either directly from the fluctuation speeds or from the Discrete Fourier Transform of the fluctuation speeds:

$$q_u \equiv \sqrt{\sum_{i=1}^{n} \left(\frac{u'_i}{\overline{U}}\right)^2} = \sqrt{\sum_{i=1}^{n} \mathcal{F}_{u,i}{}^2}$$

$$q_v \equiv \sqrt{\sum_{i=1}^{n} \left(\frac{v'_i}{\overline{U}}\right)^2} = \sqrt{\sum_{i=1}^{n} \mathcal{F}_{v,i}{}^2},$$

where $\mathcal{F}_{u,i}$ and $\mathcal{F}_{v,i}$ are the i'th elements of $\mathcal{F}_u$ and $\mathcal{F}_v$. The equivalence, which can be checked by direct substitution, is a result of Parseval's Theorem, which says that the sum of the squares of a function is equal to the sum of the squares of the function's Fourier Transform [4].

Using Parseval's Theorem provides a convenient way to define a bandpass filter with cutoff frequencies f_L and f_H:

$$q_u \text{ with } f_L \text{ to } f_H \text{ bandpass} = \sqrt{2\sum_{i=i_L}^{i_H} \mathcal{F}_{u,i}{}^2}$$

$$q_v \text{ with } f_L \text{ to } f_H \text{ bandpass} = \sqrt{2\sum_{i=i_0}^{i_H} \mathcal{F}_{v,i}{}^2},$$

where the i_L'th and i_H'th elements of f are the first to be greater than or equal to f_L and f_H, respevtively. For a highpass filter, i_H is set to $n/2 + 1$ on account of symmetry about the Nyquist frequency.

Supplemental Figure SF.1: A photograph of the wind tunnel from within the lab space. The center of the photo shows the gray contraction leading to the black test section. Only one half of the tunnel is visible; the other half is behind the white acoustic wall in the background. Photo credit: David Lentink.

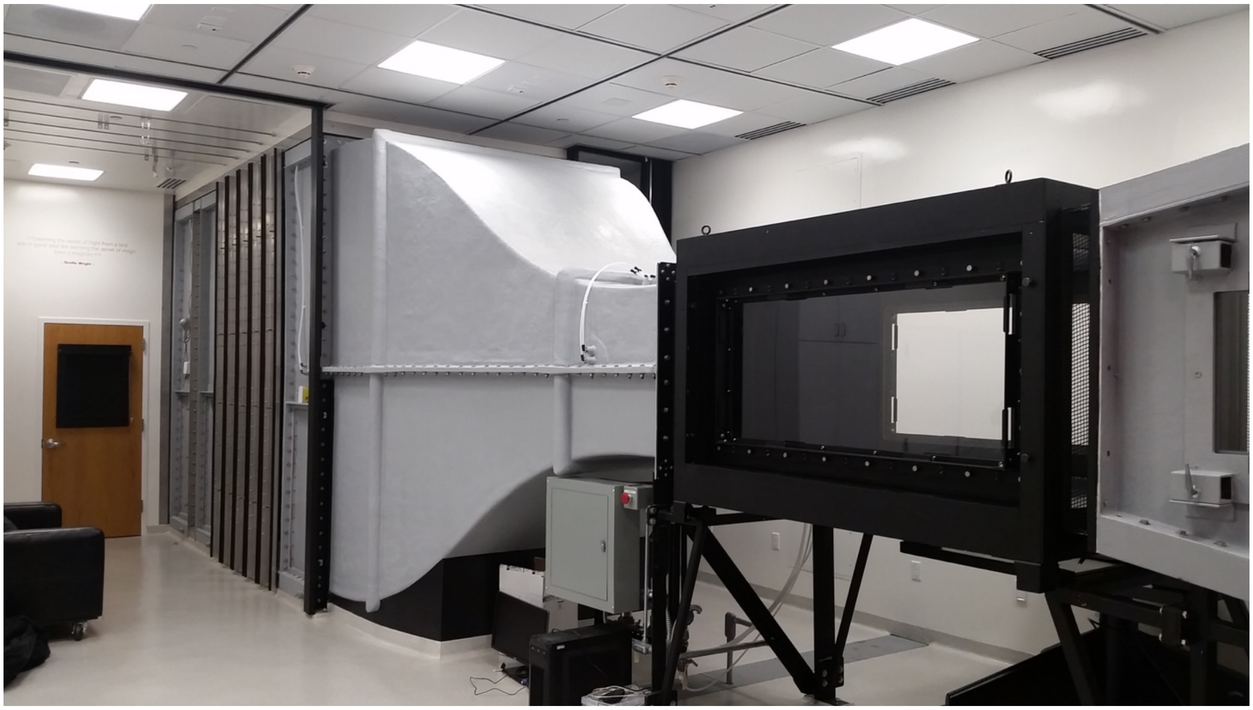

Supplemental Figure SF.2: Custom traverse for aeroacoustic measurements. (a) A steel strut (shown in yellow in this figure) can be mounted to the back end of the test section. The shadowed struts are used to show the 4 potential horizontal locations of the strut. The traverse can also be mounted such that the sting is positioned at the test section centerline (not shown). Corner 1 is not shown for clarity. (b) A schematic shows the 17 positions (as black dots) where measurements can be taken: 16 points on the 4x4 grid and 1 additional point at the test section centerline. View is from downstream looking upstream. (Note the return leg drawn in the background of this CAD model is behind a wall in the lab; see figure 1)

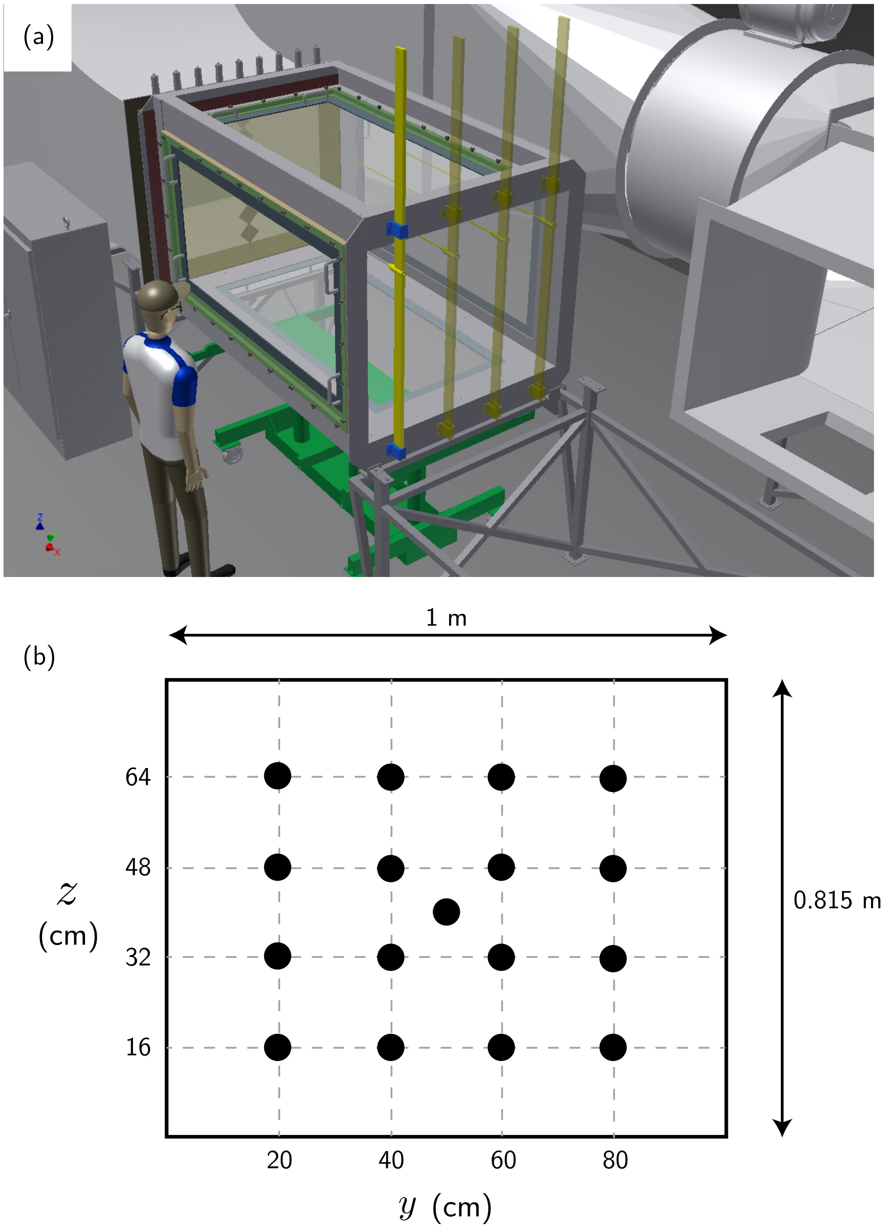

Supplemental Figure SF.3: Total pressure is uniform in the test section. The total pressure, p_T, is measured by a pitot-static probe on a 4x4 grid in the test section (black dots indicate grid points). Deviations in total pressure, Δp_T, from the average over the grid, $\overline{p_T}$, are slightly lower when the average airspeed $\overline{U}$ = 10 m/s (left panel; 1 σ of Δp_T = 0.344 Pa) compared to when $\overline{U}$ = 25 m/s (right panel; 1 σ of Δp_T = 0.970 Pa). Color indicates the relative total pressure deviation, that is, $\Delta p_T / \overline{p_T}$.

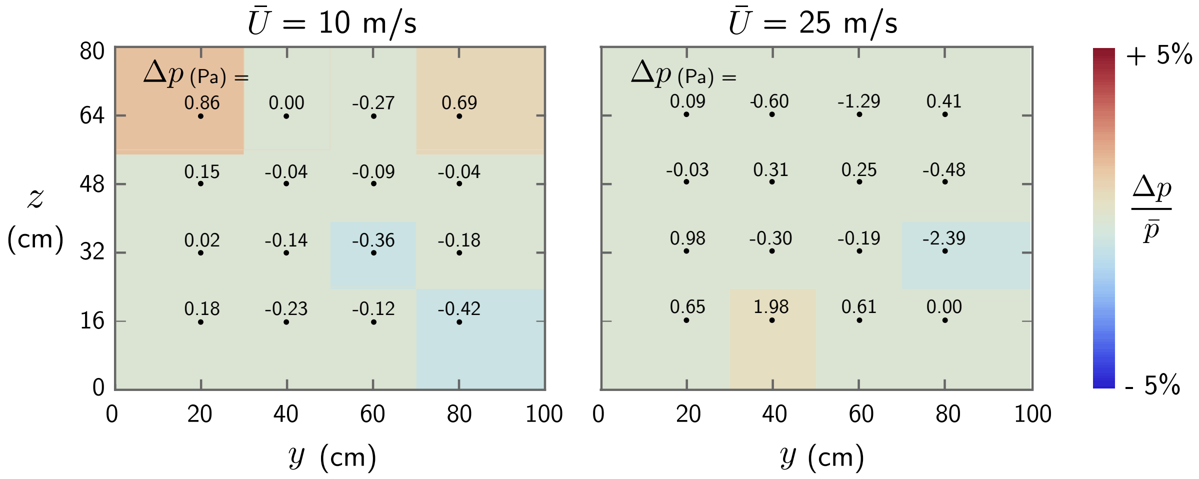

Supplemental Figure SF.4: Electrical circuit noise was removed from the velocity data before calculating turbulence intensity. The power supplied by the building creates false spikes in the Discrete Fourier Transform of the axial velocity data, $\mathcal{F}_u$. The spikes occur at the circuit frequency, 60 Hz, and the higher harmonics above (120 Hz, 180 Hz…). To remove the noise, a +/- 1Hz linear notch filter was applied at each harmonic. Values of the "original $\mathcal{F}_u$" between $60 \cdot i - 1$ Hz and $60 \cdot i + 1$ Hz (where i is a counter starting at 1) were replaced with a linear interpolation ("corrected $\mathcal{F}_u$" shown in yellow). The width of the notch filter was chosen to be wider than the peak to account for the peak widening at higher harmonics, as can already be seen in the 5th harmonic. For the sample case shown (axial velocity at centerline at 25 m/s), the odd harmonics contribute significantly more noise. This uneven contribution is typical of circuit noise; whether odd or even harmonics dominate can depend on the circuit conditions [5], so we removed all harmonics for all trials to ensure no electrical contribution to the reported turbulence intensity. The magnitude of the correction on turbulence intensity can be seen in supplemental tables ST.1 and ST.2.

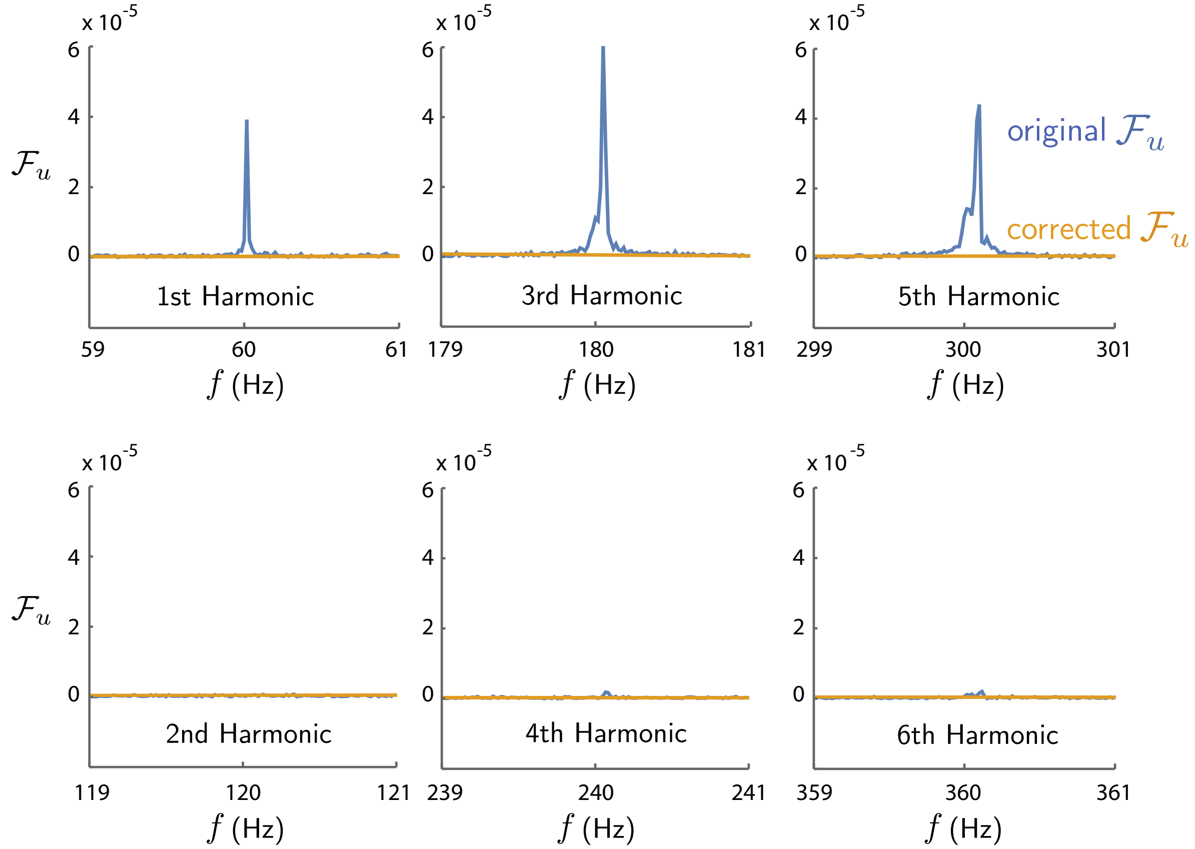

Supplemental Figure SF.5: Turbulence intensity frequency spectra are dominated by low frequencies. The frequency spectra for q_u (a) and q_v (b) are explored by plotting the median value of power over all turbulence measurements at the centerline. The energy is concentrated below the 1 kHz range, demonstrating the significance of the highpass frequency.

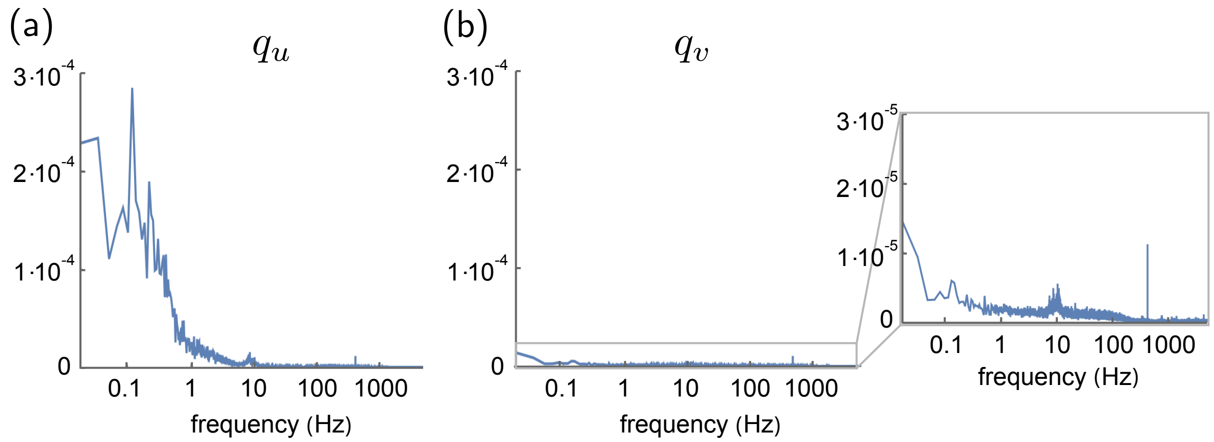

Supplemental Figure SF.6: A fiberglass mount held the microphone at the centerline of the test section. The mount was attached to an aluminum frame and came up through a slit in a custom bottom panel for the test section.

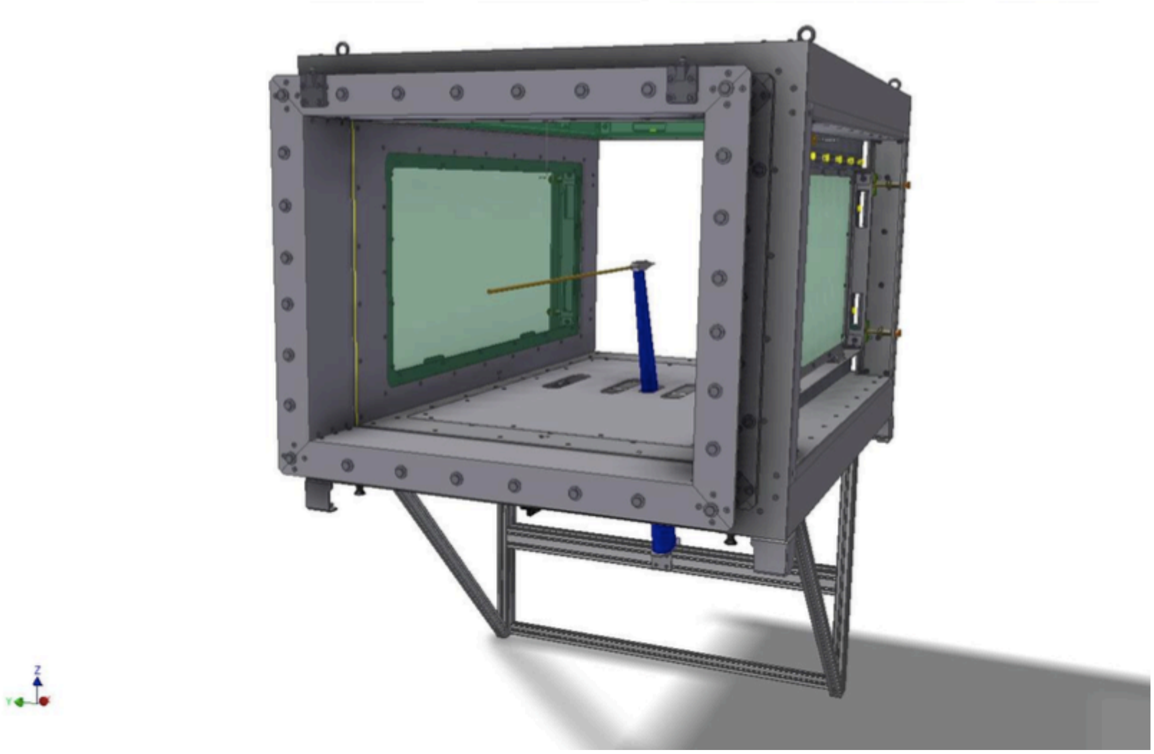

Supplemental Figure SF.7: Synthetic fur covers the acoustic turning vanes for extra noise attenuation.

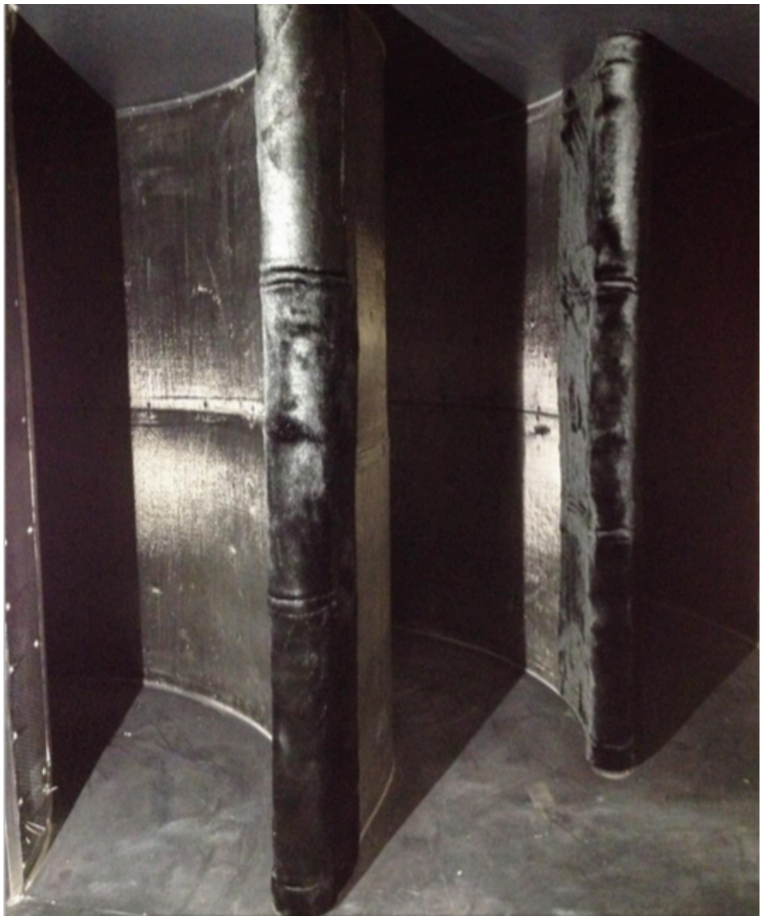

Supplemental Figure SF.8: The Active Turbulence Grid can create nonuniform velocity profiles such as jets and wakes. To demonstrate the gust-shaping abilities of the active turbulence grid, two nonuniform profiles were created: a jet, with a fast airspeed in the center compared to the sides, and a wake, with a slow airspeed in the center compared to the sides. To minimize the time to switch from uniform flow to jet/wake, the blockage was held constant between all three conditions (uniform, wake, jet). Blockage can be estimated by calculating the projected area of the vanes in each condition, but must be manually tuned based on pressure port readings since no exact solution exists for the pressure drop over a nonuniform grid. The three conditions were tuned to each produce an average flow of 10 m/s in the test section. They are defined as “Uniform” (all vanes set to +/- 37°), “Wake” (2 center vertical vanes at 90°; all other vertical vanes at 0°; horizontal vanes at +/- 44°), and “Jet” (2 center vertical vanes at 0°; all other vertical vanes at 90°; horizontal vanes at 0°). The numbers in the figure show each vane rotation in degrees. The alternating positive/negative pattern was adopted to minimize swirl in the test section. In the test, all motors were switched simultaneously from their Uniform position to either their Wake or Jet positions.

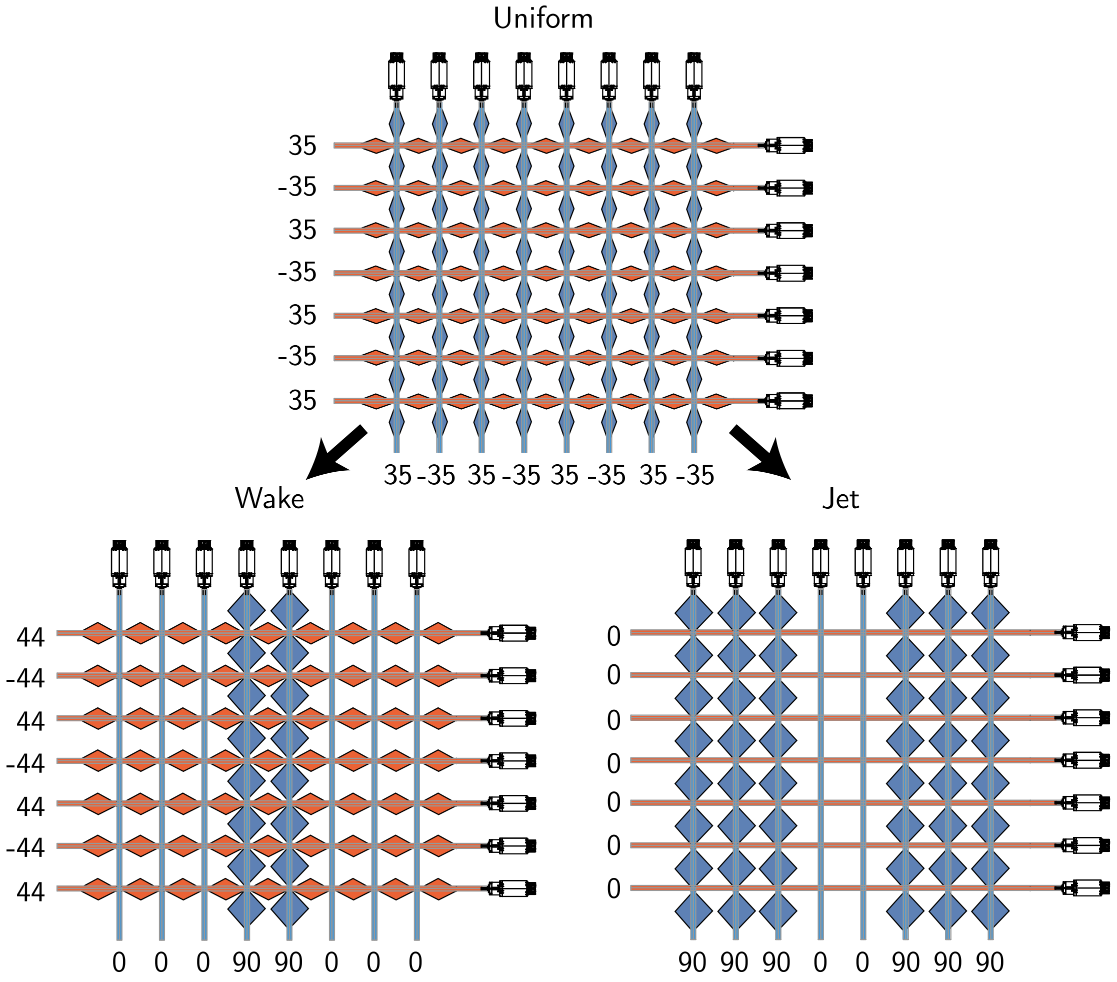

Supplemental Figure SF.9: The correction applied to temperature causes a small change in the reported temperature uniformity. The deviation in temperature from the average tunnel temperature, ΔT, is measured on a 4x4 grid in the test section (black dots indicate grid points). Unlike figure 3c in the main manuscript, here no correction was applied to the temperature data, so temporal and spatial variation cannot be separated. These data are included to demonstrate the magnitude of the correction, and are meant to be compared with figure 3c.

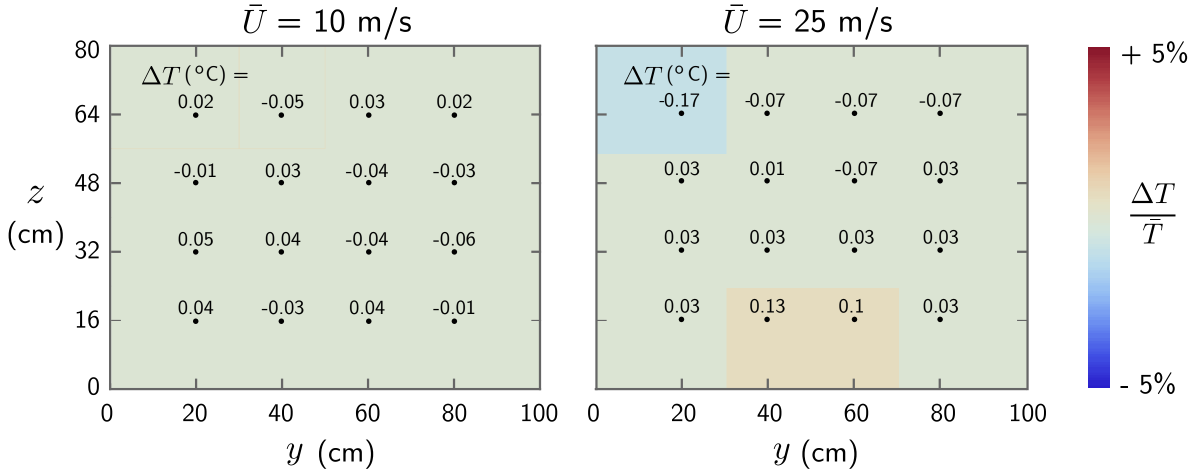

Supplemental Figure SF.10: Access hatches downstream of the test section allow easy access while working with animals in the tunnel. A 3D rendering shows how a user can stand in the airflow for quick access to the test section interior during wind tunnel operation. (Note the return leg drawn in the background of this CAD model is behind a wall in the lab; see figure 1)

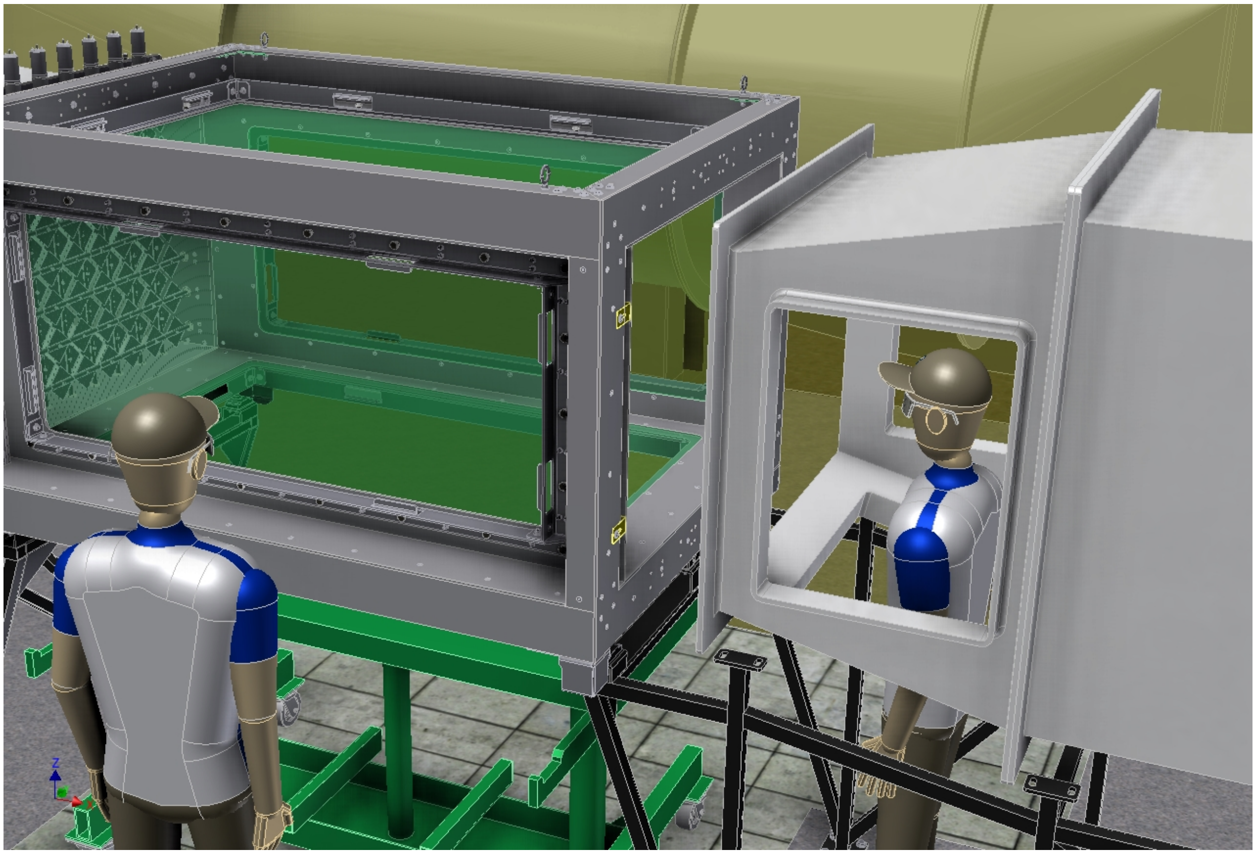

Supplemental Figure SF.11: The operating temperature changes with airspeed due to different rates of energy consumption by the fan. The maximum attainable temperature (red) varies between 20 °C and 30 °C, and the minimum attainable temperature (blue) varies between 10 °C and ~ 15 °C.

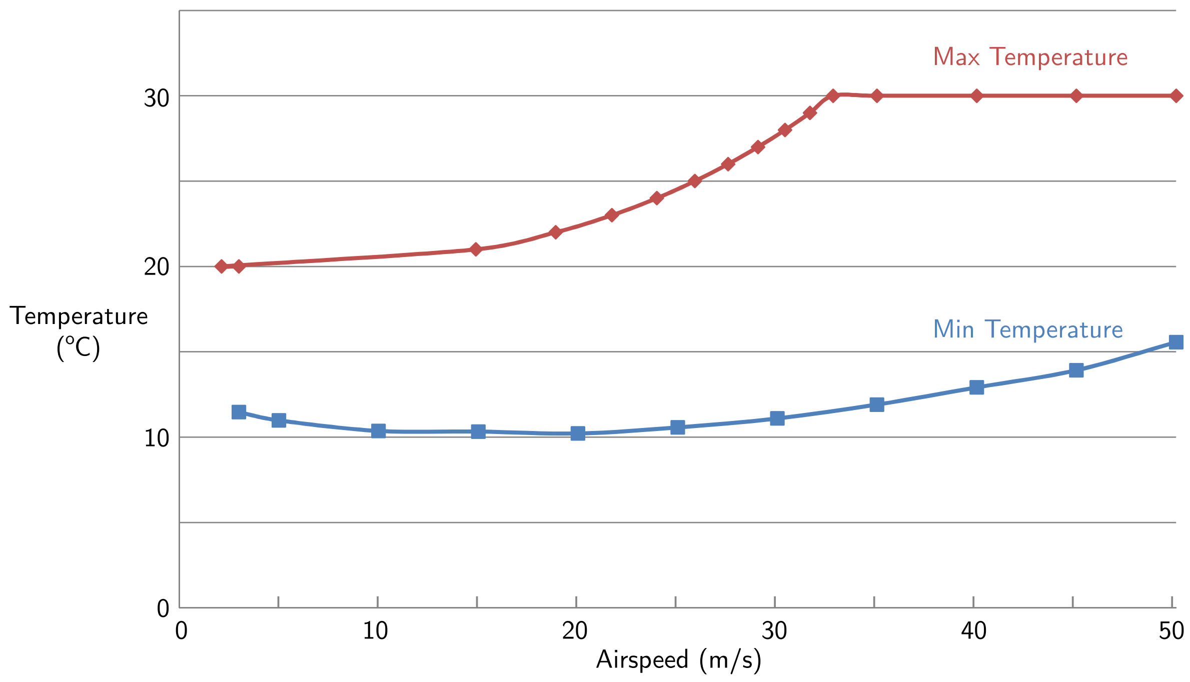

Supplemental Table ST.1

Axial turbulence intensities are significantly affected by highpass filtering. The data from the left panel of Figure 4a are shown in tabular form (values in %). The first row includes the electrical circuit noise, while the second row has been notch filtered at all harmonics of 60 Hz (supplemental figure SM.4). All subsequent rows include the 60i Hz notch filters as well as the additional filtering listed. Conditions at 20 m/s and above are based on centerline measurements, while the 10 m/s condition is the average of the four center grid points. Rows 2-7 are the curves plotted in figure 4a in the main manuscript.

	Airspeed (m/s)							
**Filter Type**	**10**	**20**	**25**	**30**	**35**	**40**	**45**	**50**
With circuit noise	0.153	0.153	0.145	0.102	0.135	0.159	0.129	0.188
60i Hz notch	0.150	0.151	0.144	0.101	0.134	0.158	0.128	0.188
0.1 Hz – 5 kHz bandpass	0.099	0.131	0.107	0.091	0.123	0.140	0.121	0.119
0.5Hz – 1kHz bandpass	0.036	0.050	0.041	0.039	0.056	0.062	0.062	0.064
1 Hz – 10 kHz bandpass	0.037	0.044	0.035	0.034	0.042	0.046	0.049	0.054
2-200 Hz bandpass	0.019	0.032	0.022	0.020	0.029	0.036	0.040	0.045
20 Hz highpass	0.028	0.022	0.021	0.021	0.020	0.019	0.018	0.019

Supplemental Table ST.2

Transverse turbulence intensities are weakly affected by highpass filtering. The transverse turbulence intensities from the right panel of figure 4a are shown in tabular form (values in %) The first row includes the electrical circuit noise, while the second row has been notch filtered at all harmonics of 60 Hz (supplemental figure SM.4). All subsequent rows include the 60i Hz notch filters as well as the additional filtering listed. Conditions at 20 m/s and above are based on centerline measurements, while the 10 m/s condition is the average of the four center grid points. Rows 2-7 are the curves plotted in figure 4a in the main manuscript.

	Airspeed (m/s)							
**Filter Type**	**10**	**20**	**25**	**30**	**35**	**40**	**45**	**50**
With circuit noise	0.038	0.034	0.030	0.027	0.028	0.029	0.028	0.028
60i Hz notch	0.033	0.032	0.027	0.026	0.026	0.028	0.027	0.027
0.1 Hz – 5kHz bandpass	0.032	0.032	0.027	0.026	0.026	0.028	0.027	0.027
0.5Hz – 1kHz bandpass	0.017	0.025	0.020	0.019	0.020	0.023	0.022	0.023
1 Hz – 10 kHz bandpass	0.031	0.032	0.027	0.026	0.026	0.027	0.027	0.027
2-200 Hz bandpass	0.009	0.022	0.017	0.016	0.017	0.020	0.019	0.020
20 Hz highpass	0.030	0.024	0.026	0.024	0.024	0.026	0.024	0.024

Supplemental Table ST.3

Overall Sound Pressure Levels are low and increase with airspeed. The Overall Sound Pressure Levels from figure 5c are shown in tabular form (values in dB). The 180 Hz highpass filter was applied using the same technique as the filters used for turbulence intensity (supplemental material SM.5). In addition to the 180 Hz highpass filter, a transfer function was used to account for amplification caused by resonance in the nosecone at frequencies below 1500 Hz.

The Sound Pressure Level (SPL) at each frequency between 0 and 1500 Hz was divided by the ratio of SPL with the nosecone to SPL without the nosecone at the corresponding frequency from figure 5a. This transfer function was inspired by a similar transfer function applied by Johansson et al. [7]. We also report values using the standard A-weighting filter [8]:

$$A(f) = 20\ \mathrm{Log}_{10}\left[\frac{12194^2 f^4}{(f^2+20.6^2)\sqrt{(f^2+107.7^2)(f^2+737.9^2)}(f^2+12194^2)}\right] + 2$$

where $A(f)$ was added to the dB value corresponding to each frequency f before calculating the OASPL.

	**Airspeed (m/s)**					
**Filter Type**	**0**	**20**	**25**	**30**	**40**	**50**
60 Hz – 30 kHz bandpass	53.2	76.4	81.8	84.4	89.9	94.5
180 Hz – 30 kHz bandpass	42.3	67.4	73.0	76.9	83.8	88.2
180 Hz – 30 kHz bandpass + Nose cone correction	42.3	62.4	68.2	72.2	80.1	83.9
A-weighting	39.0	66.0	71.4	74.7	80.5	85.1

Supplemental Table ST.4

The active turbulence grid can create turbulence intensities ranging from 12.5% to 45.1%, based on unfiltered velocity data. Intensities are higher when applying the 20 Hz highpass filter used in figure 4b (supplemental table ST.5). For these trials, vertical vanes were fully closed (the condition that maximized turbulence). The horizontal vanes oscillated with amplitude φ_V and max speed v_{MAX}. Axial turbulence values are listed in %. Rows 1-5 are used to make the array plot in figure 6b. (*: φ_H-v_{MAX} conditions beyond the physical limitations of the motors).

	**v_{MAX} (RPM)**					
**φ_V (°)**	**0**	**300**	**600**	**900**	**1200**	**1500**
0.00	12.5	12.5	12.5	12.5	12.5	12.5
8.75	12.5	18.9	21.6	21.0	22.8	21.1
17.50	12.5	25.2	26.5	30.3	32.9	31.2
26.25	12.5	26.1	32.4	40.8	41.6	45.1
35.00	12.5	30.2	43.6	43.1	*	*

Supplemental Table ST.5

The active turbulence grid can create turbulence intensities ranging from 8.1% to 16.1%, based on velocity data with a 20 Hz highpass filter. For these trials, vertical vanes were fully closed (the condition that maximized turbulence). The horizontal vanes oscillated with amplitude φ_V and max speed v_{MAX}. Axial turbulence values are listed in %. These data are not

plotted in a figure, but are included to facilitate comparisons with figure 4b, where turbulence with no active grid is reported with a 20Hz highpass filter. (*: φ_H-v_{MAX} conditions beyond the physical limitations of the motors).

	$\boldsymbol{v_{\mathrm{MAX}}}$ **(RPM)**					
$\boldsymbol{\varphi_V}$ **(°)**	**0**	**300**	**600**	**900**	**1200**	**1500**
0.00	8.1	8.1	8.1	8.1	8.1	8.1
8.75	8.1	9.6	10.3	9.7	10.6	9.8
17.50	8.1	11.4	12.6	13.1	14.2	13.4
26.25	8.1	11.4	13.4	14.7	13.6	14.7
35.00	8.1	12.3	16.1	15.2	*	*

Cited References

[1] Thompson PA. Compressible-fluid dynamics. pgs. 83, 267-268. McGraw-Hill; 1971.

[2] Bradshaw P, Pankhurst RC. The design of low-speed wind tunnels. Progress in Aerospace Sciences. 1964 Jan 1;5:1-69.

[3] Lindgren B. Flow facility design and experimental studies of wall-bounded turbulent shear-flows.

[4] Chew WC. Waves and fields in inhomogeneous media. New York: IEEE press; 1995 Jan.

[5] Vines RM, Trissell HJ, Gale LJ, O'neal JB. Noise on residential power distribution circuits. IEEE Transactions on Electromagnetic Compatibility. 1984 Nov(4):161-8.

[6] Bolton D. The computation of equivalent potential temperature. Monthly weather review. 1980 Jul;108(7):1046-53.

[7] Johansson A. A low speed wind-tunnel with extreme flow quality - Design and tests. In ICAS - 92-3.8.1, Beijing, China 1992 (pp. 1603-1611).

[8] Fletcher H, Munson WA. Loudness, its definition, measurement and calculation. Bell Labs Technical Journal. 1933 Oct 1;12(4):377-430.
